# Loss of Calreticulin Uncovers a Critical Role for Calcium in Regulating Cellular Lipid Homeostasis

**DOI:** 10.1038/s41598-017-05734-x

**Published:** 2017-07-19

**Authors:** Wen-An Wang, Wen-Xin Liu, Serpen Durnaoglu, Sun-Kyung Lee, Jihong Lian, Richard Lehner, Joohong Ahnn, Luis B. Agellon, Marek Michalak

**Affiliations:** 1 Department of Biochemistry, University of Alberta, Edmonton, Alberta T6G 2H7 Canada; 2 Department of Life Sciences, Research Institute for Natural Sciences, BK21 Plus Life Science for BDR Team, Research Institute of Natural Science, Hanyang University, Seoul, 133-791 South Korea; 3 Department of Cell Biology, University of Alberta, Edmonton, Alberta T6G 2H7 Canada; 4 School of Human Nutrition, McGill University, Ste. Anne de Bellevue, Quebec, H9X 3V9 Canada

## Abstract

A direct link between Ca^2+^ and lipid homeostasis has not been definitively demonstrated. In this study, we show that manipulation of ER Ca^2+^ causes the re-distribution of a portion of the intracellular unesterified cholesterol to a pool that is not available to the SCAP-SREBP complex. The SREBP processing pathway in ER Ca^2+^ depleted cells remained fully functional and responsive to changes in cellular cholesterol status but differed unexpectedly in basal activity. These findings establish the role of Ca^2+^ in determining the reference set-point for controlling cellular lipid homeostasis. We propose that ER Ca^2+^ status is an important determinant of the basal sensitivity of the sterol sensing mechanism inherent to the SREBP processing pathway.

## Introduction

The endoplasmic reticulum (ER)^#^ is a multifunctional intracellular organelle recognized as the single largest intracellular Ca^2+^ storage depot and is responsible for the synthesis, folding and facilitation of intracellular transport of membrane associated and secreted proteins, as well as synthesis and transport of lipids and steroids^1–3^. ER Ca^2+^ concentration is tightly regulated by ER Ca^2+^ binding proteins and transporters^3^. Many intracellular Ca^2+^-dependent signaling pathways are regulated by the ER Ca^2+^ stores^1, 3, 4^ including communication between the ER and the plasma membrane, mitochondria and nucleus^4–7^, protein synthesis/folding/secretion, and protein-protein interactions^4, 8, 9^. ER Ca^2+^ concentration and signaling are tightly regulated by binding of Ca^2+^ to ER resident proteins^2, 4^.

Calreticulin is a major Ca^2+^ binding protein in the lumen of the ER^10^ and cells deficient in calreticulin have substantially reduced ER Ca^2+^ store capacity and impaired agonist-induced Ca^2+^ release as well as delayed store-operated Ca^2+^ entry^10, 11^. Whole-body calreticulin deficiency in mice is embryonic lethal caused by impaired cardiogenesis emanating from disrupted Ca^2+^ signaling and insufficient activation of ER Ca^2+^-dependent transcriptional pathways^11–13^. Calreticulin deficient cells have impaired inositol 1,4,5-trisphosphate-dependent Ca^2+^ release^10^, inhibited calcineurin activity and nuclear translocation of NF-AT and MEF2C^11, 13^. Cardiac specific expression of constitutively active calcineurin, a Ca^2+^-dependent protein phosphatase, reverses this defect in cardiac development and rescued *Calr* ^−/−^ mice from embryonic lethality (referred to as *Calr* ^−/−^ rescued mice)^12^. An intriguing feature of the *Calr* ^−/−^ rescued mice is their extremely elevated concentration of serum lipids^12^, suggesting that disruption of ER Ca^2+^ homeostasis in non-cardiac tissues drastically perturbs lipid metabolism.

Sterol response element binding proteins (SREBPs) belong to the basic helix-loop-helix leucine zipper family of transcription factors and are synthesized as ER associated integral membrane proteins^14^. The SREBPs are thought to be master regulators of lipid homeostasis by regulating the expression of genes involved in cholesterol and triacylglycerol metabolism^15, 16^. SREBP processing is triggered by small changes in ER membrane cholesterol^17^. When ER membrane cholesterol is abundant, cholesterol binds to SREBP cleavage activating protein (SCAP), favoring SREBP retention in the ER^18–20^. However, when ER membrane cholesterol concentration is reduced, SREBPs escape the ER and undergo specific proteolytic events in the Golgi apparatus to yield the nuclear form of SREBPs (nSREBP) that induce the transcription of target genes^16^. There are two genes encoding three isoforms of SREBPs, namely SREBP-1a, SREBP-1c/ADD1 and SREBP-2. SREBP-1c and SREBP-2 preferentially regulate genes involved in triacylglycerols and cholesterol synthesis, respectively, whereas SREBP-1a is a potent regulator of both SREBP-1c and SREBP-2 target genes^15, 18^.

The discovery of hyperlipidemia in *Calr* ^−/−^ mice rescued by cardiac-specific expression of activated calcineurin^12^ suggested a possible physiological link between Ca^2+^ and lipid homeostasis. A major unanswered question is whether there is a link between ER luminal Ca^2+^ and the SREBP processing and signaling pathway. In this study we discovered that reduction of ER luminal Ca^2+^ concentration alters the distribution of intracellular cholesterol resulting in enhancement of SREBPs activation. These findings reveal a novel role for Ca^2+^ in determining the basal level of sensitivity of the sterol sensing mechanism inherent to the SREBP processing pathway.

## Results

### Calreticulin deficiency disrupts lipid homeostasis

**Figure 1:**
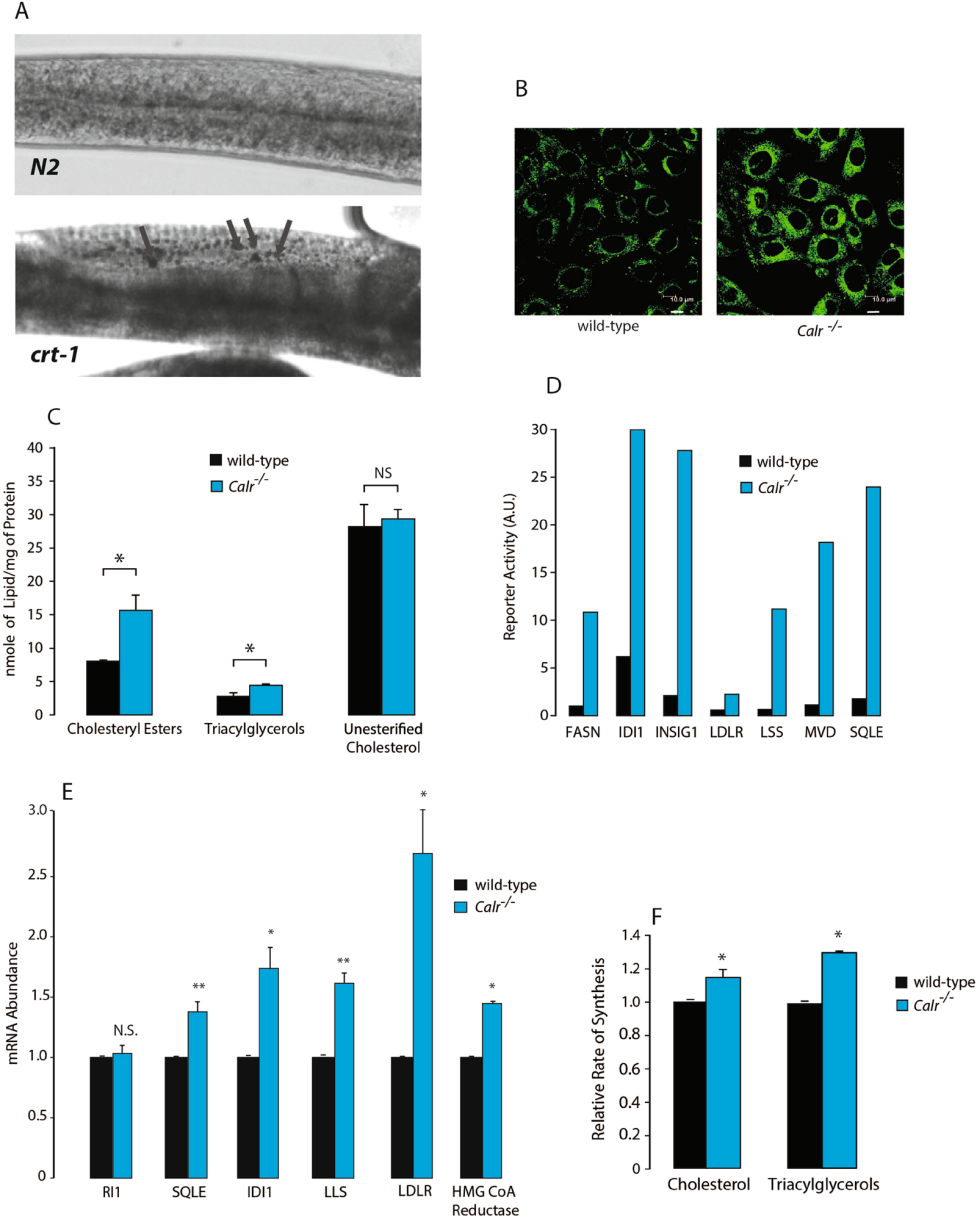
Lipids in the absence of calreticulin. (**A**) Sudan black staining of neutral lipids in wild-type (*N2*) and *crt*-*1* calreticulin deficient (*jh101*) *C*. *elegans*. Lipid droplets are indicated by the arrows. (**B**) BODIPY 505/515 staining of neutral lipids in wild-type and calreticulin-deficient (*Calr* ^−/−^) cells. (**C**) Cholesteryl esters, triacyglycerols and unesterified cholesterol levels in wild-type and *Calr* ^−/−^ cells. *Indicates statistically significant differences: cholesteryl esters, *p*-value = 0.0374; triacylglycerols, *p*-value = 0.0451. NS, not significant. Representative of 3 biological replicates. Student’s t-test was used. (**D**) SwitchGear Cholesterol Biosynthesis Pathway assay for SREBP target genes in wild-type and *Calr* ^−/−^ cells; fatty acid synthase (FASN), isopentenyl-diphosphate delta isomerase (IDI1), insulin induced gene-1 (INSIG1), low density lipoprotein receptor (LDLR), lanosterol synthase (LSS), mevalonate pyrophosphate decarboxylase (MVD) and squalene epoxidase (SQLE) Representative of 2 biological replicates. (**E**) Q-PCR analysis of replication initiator 1 (RI1), squalene (SQLE), isopentenyl-diphosphate delta isomerase (IDI1), lanosterol synthase (LSS), LDL receptor (LDLR) and HMG-CoA reductase mRNA level in wild-type and *Calr* ^−/−^ cells. Results were normalized to 18S rRNA (internal control). **Indicates statistically significant differences: SQLE in wild-type vs. *Calr* ^−/−^ cells, *p*-value = 0.0041; LSS in wild-type vs. *Calr* ^−/−^ cells, *p*-value = 0.001. *Indicates statistically significant differences: IDI1 in wild-type vs. *Calr* ^−/−^ cells, *p*-value = 0.0133; LDLR in wild-type vs. *Calr* ^−/−^ cells, *p*-value = 0.0112; HMG-CoA Reductase in wild-type vs. *Calr* ^−/−^ cells, *p*-value = 0.0001. Representative of 4 biological replicates. NS, not significant (Student’s t-test). (**F**) Analysis of rates of synthesis of cholesterol and triacyglycerols in wild-type and *Calr* ^−/−^ cells. *Indicates statistically significant differences: cholesterol, *p*-value = 0.0012; triacylglycerols, *p*-value = 0.0006 (Student’s t-test). See “Experimental Procedures” for additional details.

Calreticulin deficiency in mice is embryonic lethal^11^ but cardiac-specific expression of constitutively active calcineurin enables calreticulin-deficient mice to overcome embryonic lethality and survive to term^12^. The plasma of *Calr* ^−/−^ rescued mice appeared opaque and white (Supplementary Fig. S1) due to high lipid concentrations^12^. To verify this phenotypic outcome in a different organism, we examined calreticulin deficiency in *C*. *elegans*. Unlike in mice, calreticulin deficiency is not lethal in *C*. *elegans* ^21^. However, like in mice, the worms deficient in calreticulin revealed accumulation of neutral lipids, as reflected by intense Sudan black staining (Fig. 1A). Similarly, analysis of wild-type and *Calr* ^−/−^ mouse cells showed that the *Calr* ^−/−^ cells had a higher intensity in BODIPY 505/515 staining, consistent with the greater concentration of neutral lipids seen in the *Calr* ^−/−^ rescued mice^12^ and the calreticulin-deficient *C*. *elegans* (Fig. 1B). Biochemical analysis of wild-type and *Calr* ^−/−^ cells revealed increased cellular concentrations of triacylglycerols and cholesteryl esters but not unesterified cholesterol in *Calr* ^−/−^ cells (Fig. 1C). Thus, the impact of the absence of calreticulin, an ER Ca^2+^ buffer, on lipid homeostasis was consistent among different experimental models. This suggested a physiological linkage between Ca^2+^ and lipid homeostasis.

Since SREBPs are known regulators of genes involved in lipid homeostasis, we hypothesized that the increase in neutral lipid concentration caused by the loss of calreticulin was attributable to enhanced neutral lipid synthesis, potentially due to increased activity of SREBPs. Therefore, we used a cholesterol biosynthesis pathway screening assay system to assess the overall stimulation of SREBP responsive genes in wild-type and *Calr* ^−/−^ cells. This analysis revealed that luciferase activity driven by promoters from genes encoding fatty acid synthase (FASN), isopentenyl-diphosphate delta isomerase (IDI1), insulin induced gene-1 (INSIG-1), lanosterol synthase (LSS), mevalonate pyrophosphate decarboxylase (MVD), the low density lipoprotein receptor (LDLR) and squalene epoxidase (SQLE) were all enhanced in the absence of calreticulin (Fig. 1D). We then examined whether the increase in SREBP activity seen in *Calr* ^−/−^ cells translated to the increase of mRNA abundance of key SREBP-2 target genes. Additional quantitative RT-PCR (Q-PCR) analysis showed increases in SQLE, IDI1, LLS, LDLR and 3-hydroxy-3-methyl-glutaryl-CoA reductase (HMGR) mRNA in the *Calr* ^−/−^ cells as compared to wild-type (Fig. 1E). Measurement of *de novo* synthesis of cholesterol and triacylglycerols from acetate showed higher rates in *Calr* ^−/−^ cells than wild-type cells (Fig. 1F). Moreover, LDL uptake was functional and appeared to be enhanced in *Calr* ^−/−^ cells as compared to wild-type (Supplementary Fig. S2). Taken together, these findings demonstrated that the elimination of calreticulin gene increased nuclear SREBP (nSREBP) activity, expression of SREBP target genes and lipid synthesis.

**Figure 2:**
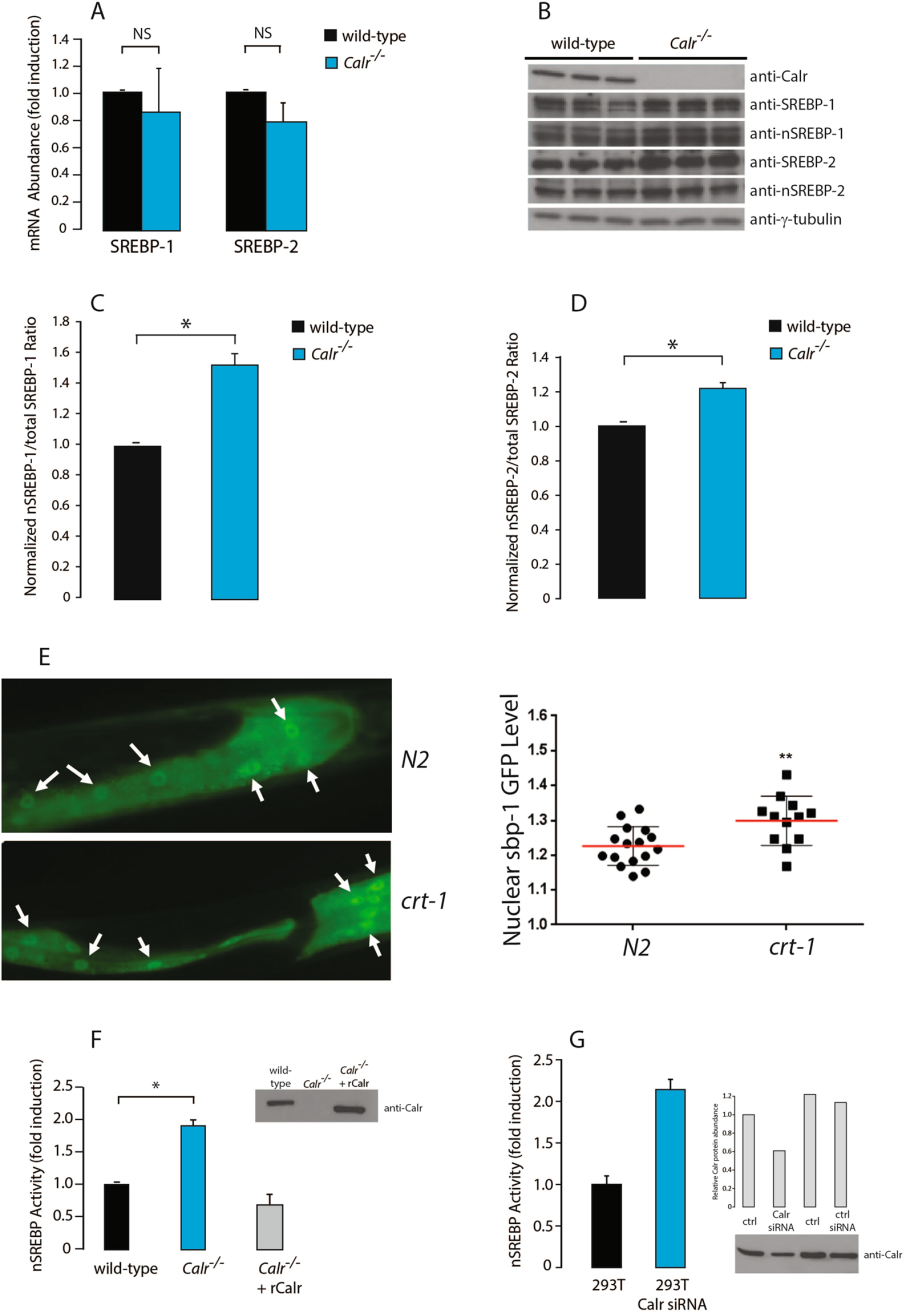
SREBP expression and processing in the absence of calreticulin. (**A**) Q-PCR quantitative analysis of total SREBP-1 and SREBP-2 mRNA abundance in wild-type and *Calr* ^−/−^ cells. Results were normalized to 18S rRNA (internal control). NS, not significant (Student’s t-test). Representative of 5 biological replicates. (**B**) Immunoblot analysis of SREBP-1, nSREBP-1, SREBP-2 and nSREBP-2 protein in wild-type and *Calr* ^−/−^ cells. Anti-γ-tubulin antibodies were used as a loading control. Representative of 3 biological replicates. (**C**,**D**) Quantitative analysis of immunoblots showing the ratio of nuclear to total SREBP-1 (**C**) and total SREBP-2 (**D**) in wild-type and *Calr* ^−/−^ cells. The value for the total is the sum of precursor and nuclear forms of SREBP. *Indicates statistically significant differences: SREBP-1, *p*-value = 0.0017 (Student’s t-test); SREBP-2, *p*-value = 0.0218 (Student’s t-test). Representative of 3 biological replicates. (**E**) GFP:SBP-1 accumulation in the intestinal nucleus (arrows) in wild-type *N2* and calreticulin deficient *crt*-*1 C*. *elegans*. Worms expressing GFP:SBP-1 driven by *sbp*-*1* promoter are shown. The average ratio of fluorescence in the nucleus and cytoplasm was calculated in each worm, and scatter-plotted (*right panel*). **Indicates statistically significant differences: *p*-value < 0.01 (Student’s t-test). Representative of 3 biological replicates. (**F**) Wild-type, *Calr* ^−/−^ cells and *Calr* ^−/−^ cells transfected with a calreticulin expression vector (*Calr* ^−/−^ +rCalr) were co-transfected with the SRE luciferase reporter plasmid followed by luciferase assay. *Indicates statistically significant difference: *p*-value = 0.0006 (Student’s t-test). Representative of 3 biological replicates. rCalr, recombinant calreticulin. Inset: immunoblot analysis with anti-Calr antibodies. (**G**) HEK293T cells were transfected with calreticulin specific siRNA or scrambled siRNA and with the SRE luciferase reporter plasmid. Representative of 3 biological replicates. Inset: immunoblot analysis with anti-Calr antibodies. Calr, calreticulin. Ctrl, control. The images of (**B**) shown are cropped. The full-length gels/blots are shown in Fig. S8. See “Experimental Procedures” for additional details.

To determine the basis for increased nSREBP activity resulting from the loss of calreticulin, we examined SREBP gene expression at the mRNA and protein levels. Q-PCR analysis showed no differences in the abundance of SREBP-1 or SREBP-2 mRNA (Fig. 2A) between wild-type and *Calr* ^−/−^ cells. However, consistent with the increased nSREBP activity reported by the SwitchGear assay, there was increased ratio of the nuclear to total abundance of SREBP-1 and SREBP-2 in *Calr* ^−/−^ cells (Fig. 2B,C,D). To determine if the same effect is evident in calreticulin-deficient worms, we created a GFP-tagged *sbp*-*1*, the *C*. *elegans* version of SREBP. Analysis of GFP-SBP-1 distribution revealed higher nuclear localization in the calreticulin-deficient worms (Fig. 2E). These findings demonstrated that calreticulin deficiency affected SREBP processing.

Consistent with increased processing of SREBP, the nSREBP activity was increased in *Calr* ^−/−^ cells (Fig. 2F). Importantly, ectopic expression of calreticulin in *Calr* ^−/−^ cells decreased nSREBP activity to a level comparable to that of wild-type cells (Fig. 2F). Conversely, calreticulin-specific siRNA-mediated silencing of calreticulin in HEK293T cells increased nSREBP activity (Fig. 2G) reproducing the effect seen in *Calr* ^−/−^ cells (Fig. 2F). Taken together, the elimination or attenuation of calreticulin gene expression in both mammalian cells and in *C*. *elegans* consistently led to increased nSREBP activity.

### SREBP processing and responsiveness to changes in cellular cholesterol status remain functional in the absence of calreticulin

**Figure 3:**
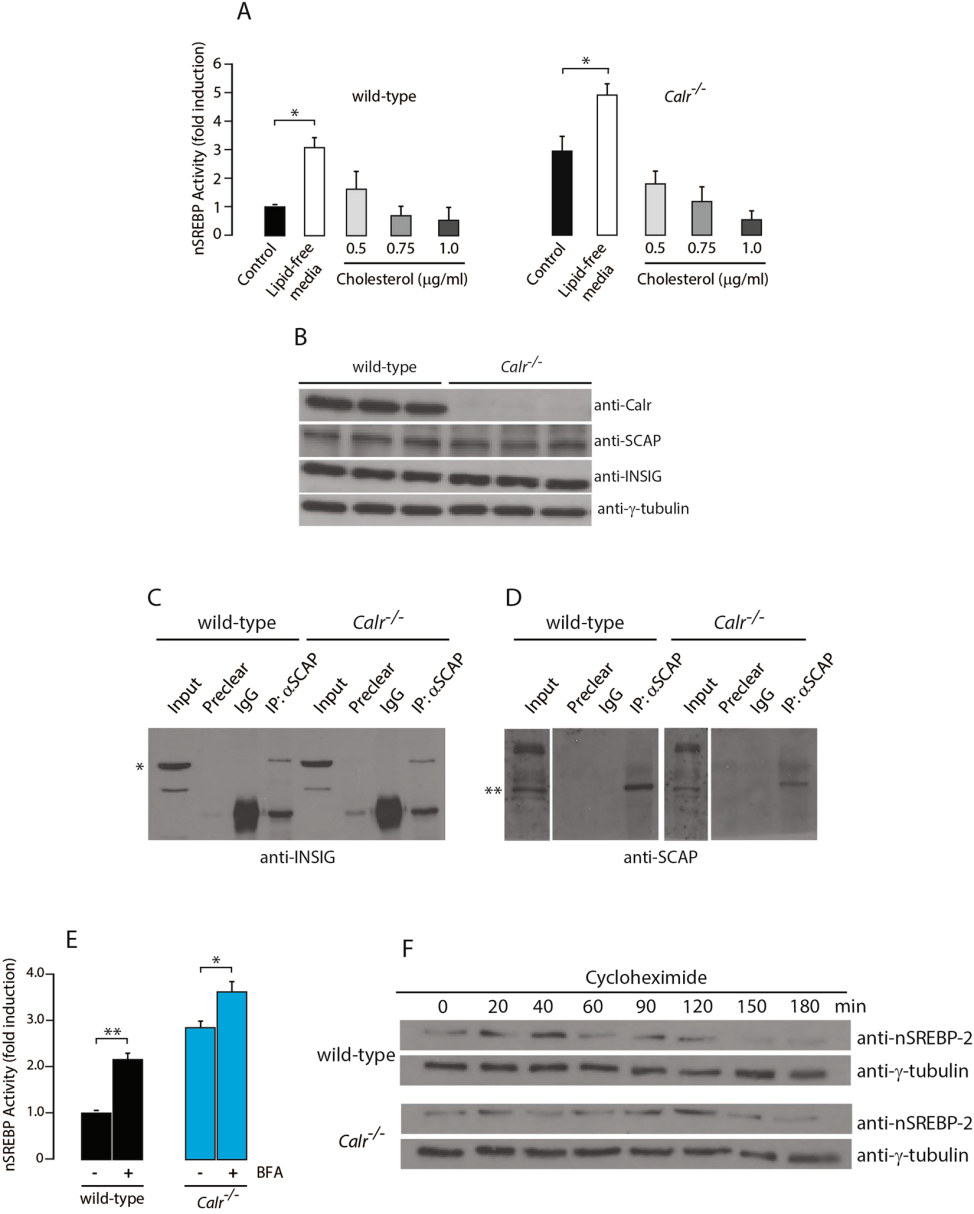
SREBP complex in the absence of calreticulin. (**A**) nSREBP activity in wild-type (*left panel*) and *Calr* ^−/−^ (*right panel*) cells with normal media, lipid-free media and lipid-free media plus 0.5, 0.75, and 1.0 µg/ml of cholesterol. *Indicates statistically significant differences: wild-type cells with normal vs. lipid-free media, *p*-value < 0.05 (ANOVA); *Calr* ^−/−^ cells with normal vs. lipid-free media, *p*-value < 0.05 (ANOVA). Representative of 3 biological replicates. (**B**) Wild-type and *Calr* ^−/−^ cells were probed with anti-Calr, anti-SCAP and anti-INSIG antibodies. γ-tubulin was used as a loading control. Representative of 3 biological replicates. (**C**,**D**) Immunoprecipitation (*IP*) assay was carried out on confluent wild-type and *Calr* ^−/−^ cells with anti-SCAP antibody. Immunoblot analysis was carried out with anti-INSIG (**C**) and anti-SCAP (**D**) antibodies. *Indicates the location of INSIG protein band. **indicates the location of SCAP protein band. Representative of 3 biological replicates. (**E**) nSREBP activity in wild-type and *Calr* ^−/−^ cells treated with Brefeldin A (BFA) (1 µg/ml). **Indicates statistically significant differences: wild-type vs. *Calr* ^−/−^ cells in control conditions, *p*-value = 0.0001 (Student’s t-test); wild-type cells in control vs. BFA treatment, *p*-value = 0.0004 (Student’s t-test). *Indicates statistically significant difference: *Calr* ^−/−^ cells in control vs. BFA treatment, *p*-value = 0.0331 (Student’s t-test). Representative of 3 biological replicates. (**F**) Immunoblot analysis of wild-type and *Calr* ^−/−^ cells probed with anti-nSREBP-2 antibody. γ-tubulin was used as a loading control. Cells were treated with cycloheximide for 0, 20, 40, 60, 90, 120, 150 and 180 min. Representative of 3 biological replicates. The images of (**B**,**C**,**D**,**F**) shown are cropped. The full-length gels/blots are shown in Fig. S9. See “Experimental Procedures” for additional details.

The conundrum that remained is how the loss of calreticulin leads to increased abundance of nSREBP while the intracellular concentration of unesterified cholesterol (Fig. 1C) did not differ between wild-type and *Calr* ^−/−^ cells. The SREBP processing pathway is sensitive to the availability of ER membrane cholesterol and this is mediated through SCAP, a sterol sensor localized to the ER membrane that forms a complex with SREBP^15, 22^. Therefore, we assessed if SCAP function was altered in *Calr* ^−/−^ cells. Wild-type and *Calr* ^−/−^ cells were incubated with lipid-free media supplemented with increasing concentrations of cholesterol and then analyzed for nSREBP activity. The nSREBP activity was increased in both wild-type and *Calr* ^−/−^ cells grown in a lipid-free media and there was a corresponding decrease in nSREBP activity with increasing concentrations of cholesterol (Fig. 3A). Furthermore in *Calr* ^−/−^ cells there was a corresponding increase in *de novo* cholesterol synthesis from acetate following removal of cholesterol source in the growth media (Supplementary Fig. S3). These experiments demonstrated that the loss of calreticulin did not abolish the functionality of SCAP and the SREBP pathway and that the responsiveness of the SREBP pathway at the level of sterol sensing remained fully operational in the absence of calreticulin.

In cholesterol excess conditions, the SCAP/SREBP complex is retained in the ER by interaction with INSIG^23^. Thus, we asked whether the INSIG-SCAP interaction was perturbed in the absence of calreticulin. Immunoblot analysis showed no difference in the abundance of SCAP or INSIG proteins between wild-type and *Calr* ^−/−^ cells (Fig. 3B). SCAP was co-immunoprecipitated with INSIG in both wild-type and *Calr* ^−/−^ cells (Fig. 3C,D), indicating that the INSIG-SCAP complex formation was not affected by the absence of calreticulin. Taken together these findings indicated that INSIG and SCAP abundance (Fig. 3B) and complex formation (Fig. 3C) were not affected by the loss of calreticulin.

A critical step in SREBP processing is the translocation of SREBPs to the Golgi, where it is proteolytically processed by site-1- and site-2-proteases (S1P and S2P), respectively. We therefore asked whether the increased SREBP activity seen in the absence of calreticulin is attributable to changes in proteolytic processing due to either retention of S1P and S2P in the ER or hyperactivity of these proteases in the Golgi. We used Brefeldin A, a known inhibitor of anterograde transport, to test if SREBP activation in wild-type and *Calr* ^−/−^ cells was sensitive to ER retention of S1P and S2P proteases. In accordance to what was previously reported^22^, Brefeldin A treatment of wild-type cells led to proteolytic processing of SREBP in the absence of lipid depletion (Fig. 3E). Similarly, there was an increase in SREBP processing in Brefeldin A treated *Calr* ^−/−^ cells (Fig. 3E), thus indicating that the increased abundance of nSREBP in *Calr* ^−/−^ cells was not attributable to ER retention of the S1P and S2P. Next we used S1P specific siRNAs, the first protease involved in processing of SREBP, to probe the activity of this protease in the processing of SREBP in the absence of calreticulin. Silencing of S1P reduced SREBP processing in *Calr* ^−/−^ cells to a level comparable to the wild-type cells (Supplementary Fig. S4). We considered that increased activity of nSREBP in *Calr* ^−/−^ cells may be due to a decrease in the rate of nSREBP degradation. To test this, cells were treated with cycloheximide followed by immunoblot analysis of nSREBP-2. We found that the rate of degradation of nSREBP-2 was the same in wild-type and *Calr* ^−/−^ cells (Fig. 3F). Taken together these observations indicated that S1P and S2P proteases were functional in wild-type and *Calr* ^−/−^ cells. Furthermore, the ability of INSIG/SCAP/SREBP to form complexes, the transport of the SREBP complex from the ER to Golgi and the degradation of nSREBPs were all fully functional in the absence of calreticulin. These critical steps in SREBP processing in response to ER membrane cholesterol depletion were not disrupted in the absence of calreticulin.

**Figure 4:**
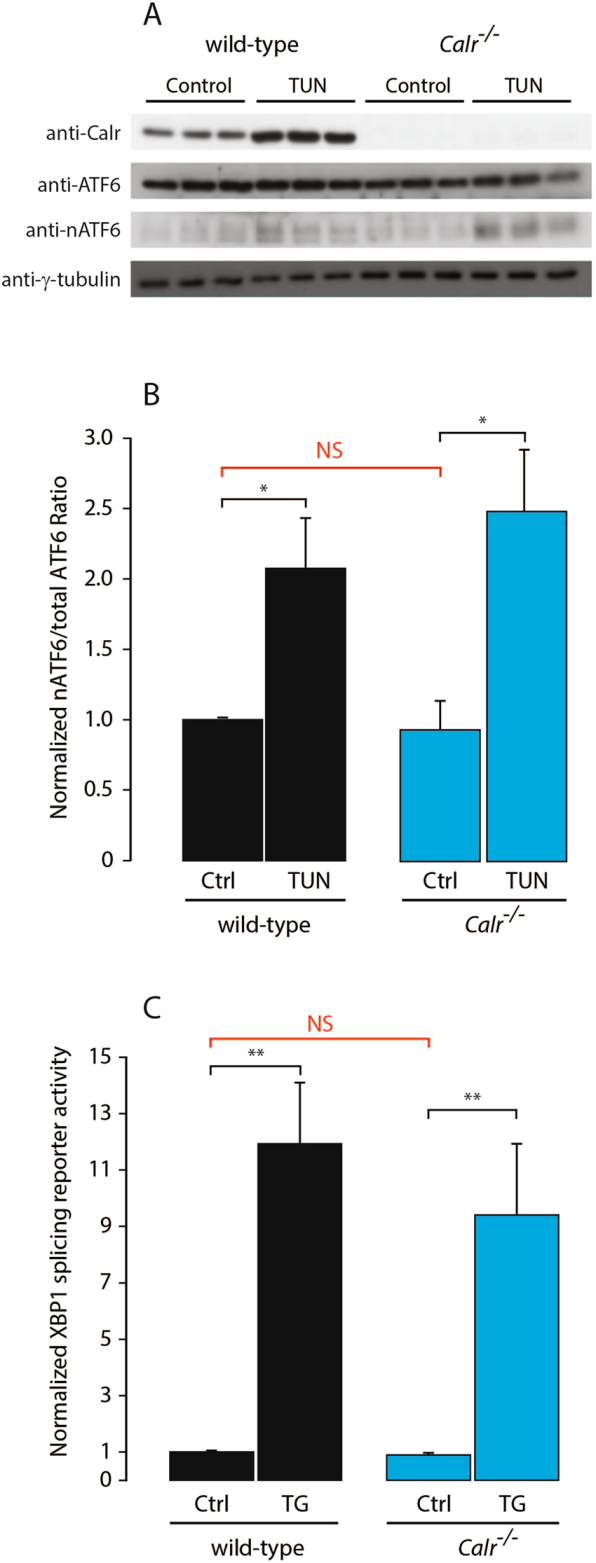
Unfolded protein response (UPR) in *Calr* ^−/−^ cells. (**A**) Immunoblot analysis of wild-type and *Calr* ^−/−^ cells probed with anti-calreticulin (Calr), anti-ATF6, anti-nuclear ATF6 (nATF6) antibodies. γ-tubulin was used as a loading control. Cells were treated without or with 5 ng/ml of tunicamycin (TUN). (**B**) Quantitative analysis of immunoblots showing the ratio of nuclear to total ATF6. *Indicates statistically significant differences: wild-type cells vs. wild-type cells treated with TUN, *p*-value = 0.0393 (Student’s t-test); *Calr* ^−/−^ cells vs. *Calr* ^−/−^ cells treated with TUN, *p*-value = 0.0265 (Student’s t-test). Representative of 3 biological replicates. NS, not significant. Ctrl, control. See “Experimental Procedures” for additional details. (**C**) XBP1-luciferase reporter activity in wild-type and *Calr* ^−/−^ cells and in cells treated without or with 1 µM thapsigargin (TG). **Indicates statistically significant differences: wild-type cells vs. wild-type cells treated with TG, *p*-value = 0.0005 (Student’s t-test); *Calr* ^−/−^ cells vs. *Calr* ^−/−^ cells treated with TG, *p*-value = 0.0051 (Student’s t-test). Representative of 6 biological replicates. NS, not significant. Ctrl, control. The images of (**A**) shown are cropped. The full-length gels/blots are shown in Fig. S10. See “Experimental Procedures” for additional details.

SREBP signaling can be induced upon activation of the unfolded protein response (UPR), an ER stress coping response^24–26^. Therefore, we measured ATF6 protein processing and XBP1 mRNA splicing to test whether *Calr* ^−/−^ cells had increased UPR and to determine if this could have contributed to nSREBP activation. We found that the abundance of nATF6 and the splicing of XBP1 mRNA were not affected by the absence of calreticulin (Fig. 4) and thus indicated that there was no activation of the UPR in *Calr* ^−/−^ cells. Importantly, induction of UPR increased both nuclear ATF6 (nATF6) abundance and XBP1 mRNA splicing in both wild-type and *Calr* ^−/−^ cells (Fig. 4), illustrating that the UPR was fully functional in the absence of calreticulin. We concluded that the increased activity of nSREBP in the absence of calreticulin was not due to activation of the UPR.

### ER Ca^2+^ modifies the ability of the SREBP pathway to sense intracellular cholesterol

**Figure 5:**
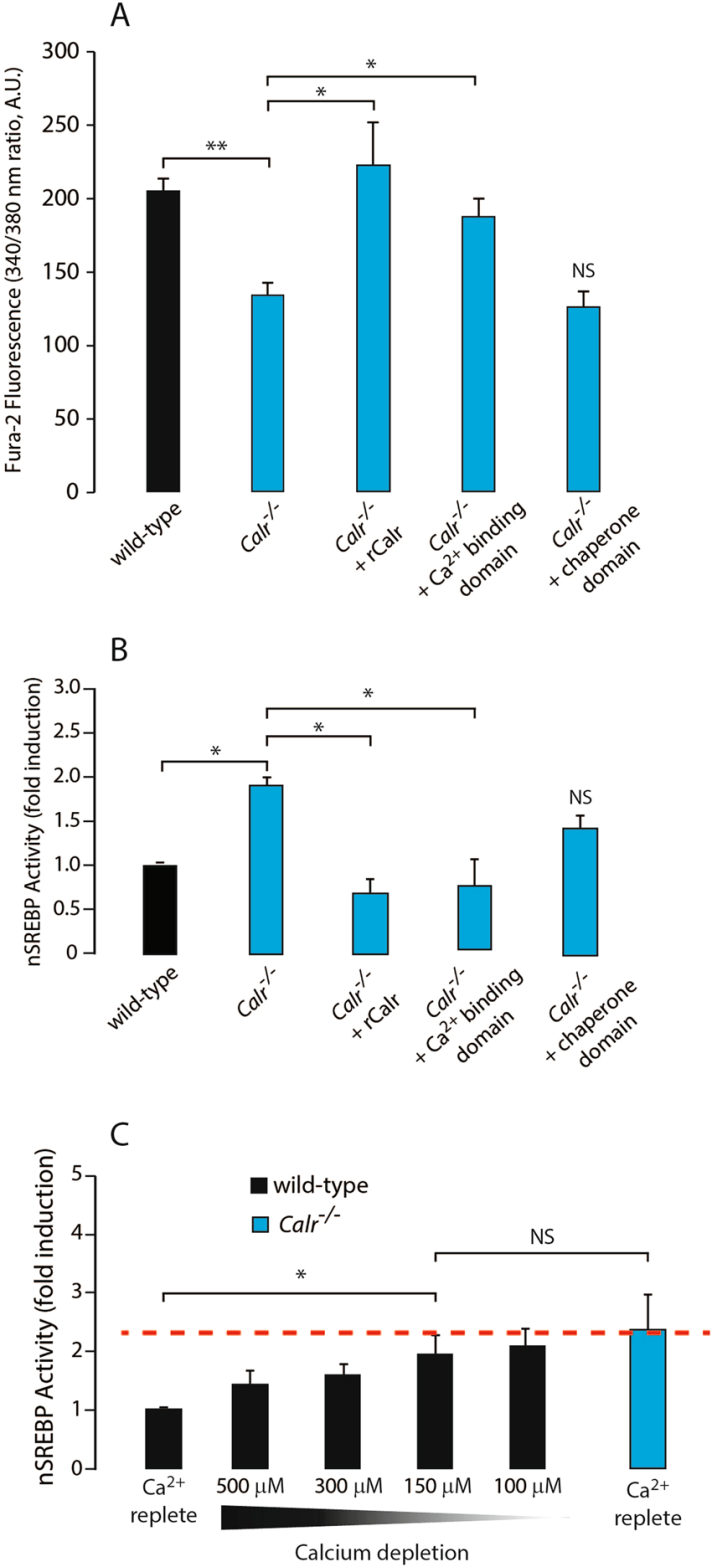
Changes in ER Ca^2+^ alters nSREBP activity. (**A**) ER luminal Ca^2+^ (thapsigargin releasable pool of Ca^2+^) in wild-type cells, *Calr* ^−/−^ cells and *Calr* ^−/−^ cells expressing full length recombinant calreticulin (*Calr*), the calreticulin Ca^2+^ binding domain or the calreticulin chaperone domain. **Indicates statistically significant differences: wild-type vs. *Calr* ^−/−^ cells, *p*-value < 0.05 (ANOVA). *Indicates statistically significant differences: *Calr* ^−/−^ vs. *Calr* ^−/−^ cells expressing rCalr, *p*-value < 0.05 (ANOVA); *Calr* ^−/−^ vs. *Calr* ^−/−^ cells expressing calreticulin Ca^2+^ binding domain, *p*-value < 0.05 (ANOVA). Representative of 3 biological replicates. NS, not significant. (**B**) nSREBP activity in wild-type cells, *Calr* ^−/−^ cells and *Calr* ^−/−^ cells expressing full length recombinant calreticulin (*rCalr*), the calreticulin chaperone domain or the calreticulin Ca^2+^ binding domain. *Indicates statistically significant differences: wild-type vs. *Calr* ^−/−^ cells, *p*-value < 0.05 (ANOVA); *Calr* ^−/−^ vs. *Calr* ^−/−^ cells expressing full length recombinant calreticulin (*rCalr*), *p*-value < 0.05 (ANOVA); wild-type vs. *Calr* ^−/−^ expressing calreticulin Ca^2+^ binding domain, *p*-value < 0.05 (ANOVA). Representative of 3 biological replicates. NS, not significant. (**C**) nSREBP activity in wild-type cells exposed to decreasing extracellular Ca^2+^ concentration to deplete ER Ca^2+^ stores. Ca^2+^ replete represents 2.17 mM extracellular Ca^2+^ concentration. *Indicates statistically significant differences, wild-type vs. *Calr* ^−/−^ cells in control conditions, *p*-value < 0.05 (ANOVA); wild-type cells in control vs. 150 µM Ca^2+^, *p*-value < 0.05 (ANOVA). Representative of 7 biological replicates. NS, not significant. See “Experimental Procedures” for additional details.

Calreticulin deficiency leads to ~50% reduction of the ER luminal Ca^2+^ content^10^. We treated wild-type and *Calr* ^−/−^ cells with thapsigargin, an inhibitor of SERCA^27^, to deplete the ER luminal Ca^2+^ as a measure of the ER Ca^2+^ content (thapsigargin releasable Ca^2+^). In agreement with our earlier observations^10^ there was a reduction of ER luminal Ca^2+^ in *Calr* ^−/−^ cells and this was normalized by expression of full length recombinant calreticulin or its Ca^2+^ binding domain, but not with the calreticulin chaperone domain^10^ (Fig. 5A). Furthermore, when we measured nSREBP activity, only the ectopic expression of the full length, or more importantly just the Ca^2+^ binding domain of calreticulin, in *Calr* ^−/−^ cells returned nSREBP activity to the level seen in the wild-type cells (Fig. 5B), the condition when the ER Ca^2+^ content was restored to that seen in wild-type cells^10^ (Fig. 5A).

To see if reduction of ER Ca^2+^ in wild-type cells could reproduce the enhanced SREBP activity observed in *Calr* ^−/−^ cells, we evaluated the nSREBP activity in wild-type cells under reduced Ca^2+^ conditions. We gradually reduced ER luminal Ca^2+^ in wild-type cells by chelating extracellular concentration of Ca^2+^ ^28^. Decreasing extracellular Ca^2+^ concentration from 500 µM to 100 µM progressively and concomitantly increased nSREBP activity in wild-type cells (Fig. 5C) whereas the same treatment had no effect on *Calr* ^−/−^ cells (Supplementary Fig. S5A). At 150 µM extracellular Ca^2+^ concentration nSREBP activity in the wild-type cells was no longer different from *Calr* ^−/−^ cells (Fig. 5C). Similar to what we observed in *Calr* ^−/−^ cells (Fig. 2B,C), there was an increase in ratio of nuclear to total abundance of SREBP-1 and SREBP-2 in wild-type cells exposed to 150 µM extracellular Ca^2+^ (condition of reduced ER Ca^2+^) (Supplementary Fig. S5B,C,D). This demonstrated that lowering Ca^2+^ concentration in wild-type cells, without changing exogenous cholesterol availability, was sufficient to reproduce the increased nSREBP activity seen in *Calr* ^−/−^ cells. Taken together, these results demonstrated that the reduction of ER luminal Ca^2+^ content is responsible for the increased abundance of nSREBP seen in *Calr* ^−/−^ cells.

### ER Ca^2+^ dictates the intracellular distribution of cholesterol

**Figure 6:**
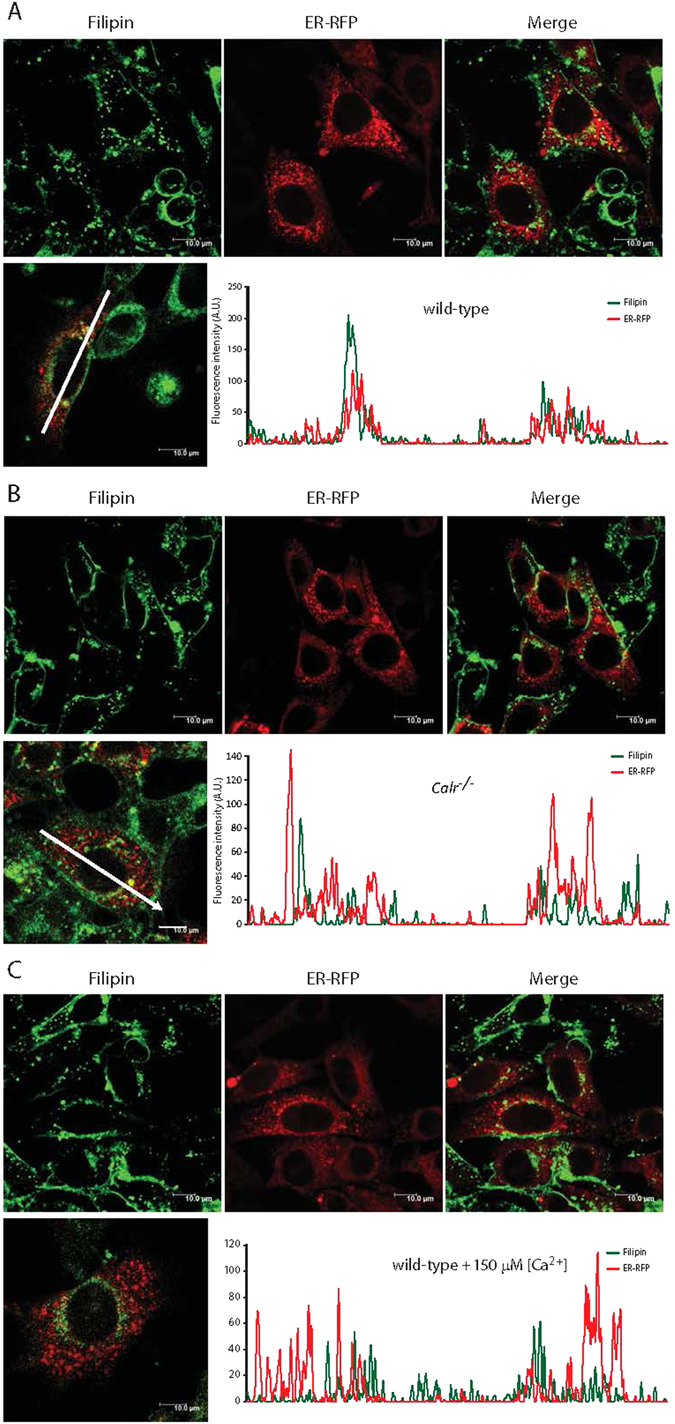
Intracellular distribution of cholesterol in wild-type and calreticulin-deficient cells. Wild-type cells (**A**) *Calr* ^−/−^ cells (**B**) and wild-type cells treated with 150 µM Ca^2+^ (**C**) expressing ER-targeted red fluorescent protein (ER-RFP) were stained with filipin. A representative experiment of 3 independent analyses is presented. Wild-type cells Pearson’s coefficient = 0.212 ± 0.013, *Calr* ^−/−^ cells Pearson’s coefficient = 0.116 ± 0.026 (n = 15), and wild-type cells in 150 µM Ca^2+^ Pearson’s coefficient = 0.118 ± 0.025 (n = 15). Graphic representation of overlap of filipin and ER-RFP signals is presented in the Figure. The arrows indicate the direction of the scan represented in the graph. See “Experimental Procedures” for additional details.

**Figure 7:**
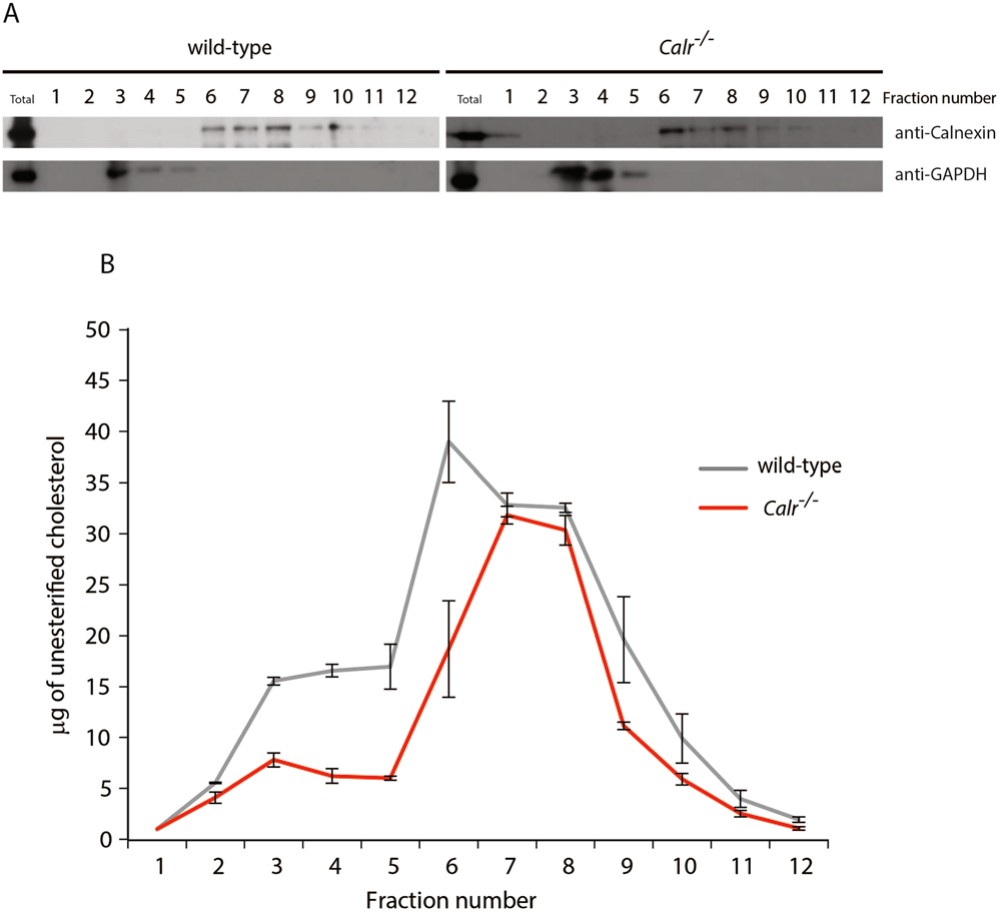
Distribution of unesterified cholesterol in wild-type and calreticulin-deficient subcellular fractions. (**A**) Immunoblot analysis of wild-type and *Calr* ^−/−^ subcellular fractions probed with anti-calnexin and anti-GAPDH antibodies. Representative of 3 biological replicates. (**B**) Distribution of unesterified cholesterol in the subcellular fractions of wild-type and *Calr* ^−/−^ cells. Representative of 3 biological replicates. The images of (**A**) shown are cropped. The full-length gel/blots are shown in Fig. S11. See “Experimental Procedures” for additional details.

Since the concentration of total intracellular unesterified cholesterol did not differ between wild-type and *Calr* ^−/−^ cells (Fig. 1C), we asked what caused the increased nSREBP activity when ER Ca^2+^ was reduced. To address this we examined the intracellular distribution of unesterified cholesterol. First we stained cells expressing ER targeted red fluorescent protein (ER-RFP) with anti-SREBP-2 antibodies and found that SREBP-2 overlaps with the ER marker in both wild-type and *Calr* ^−/−^ cells (Supplementary Fig. S6, Pearson’s Coefficient = 0.231 ± 0.035 and Pearson’s Coefficient = 0.291 ± 0.038, respectively [n = 15]). Next we used filipin, a fluorescent polyene antibiotic, to detect the localization of unesterified cholesterol within cells^29, 30^. In the wild-type cells, filipin staining co-localized with the ER-RFP marker (Fig. 6A, Pearson’s Coefficient for the wild-type cells: 0.212 ± 0.013 [n = 15]), and analysis of the filipin and ER-RFP signals across these cells revealed excellent overlap of the two signals (Fig. 6A) indicating that cholesterol was co-localized in the ER. In contrast, there was substantially less overlap of the filipin signal with the ER-RFP marker in *Calr* ^−/−^ cells (Fig. 6B, Pearson’s Coefficient for the *Calr* ^−/−^ cells: 0.116 ± 0.026 [n = 15]). Next we exposed wild-type cells to 150 µM extracellular Ca^2+^ to reduce their ER luminal Ca^2+^ ^28^, a condition resulting in increased nSREBP activity (Fig. 5C). The cells were then analyzed to determine whether the reduced ER Ca^2+^ content also affected the distribution of cholesterol in wild-type cells. Remarkably, in wild-type cells exposed to 150 µM Ca^2+^ the overlap of cholesterol with ER-RFP marker was substantially reduced (Fig. 6C, Pearson’s Coefficient for the wild type cells with 150 µM Ca^2+^: 0.118 ± 0.025 [n = 15]) reminiscent of the pattern exhibited by *Calr* ^−/−^ cells cultured under normal conditions (Fig. 6B). Next, we fractionated wild-type and *Calr* ^−/−^ cells on an Optiprep gradient and determined distribution of unesterified cholesterol within each of the fractions (Fig. 7). Immunoblot analysis revealed that fractions 3 to 5 were positive for glyceraldehyde 3-phosphate dehydrogenase (GAPDH), a marker of cytosol and fractions 6 to 10 corresponded to the ER as indicated by the presence of calnexin (Fig. 7A). In agreement with the distribution of unesterified cholesterol revealed by filipin staining (Fig. 6), the distribution of unesterified cholesterol detected in subcellular fractions obtained from wild-type and *Calr* ^−/−^ cells were distinct (Fig. 7A). In the wild-type cells the peak of unesterified cholesterol within calnexin positive fractions (i.e., ER fractions) was found in fraction 6 (Fig. 7B). In contrast, the peak of unesterified cholesterol within calnexin positive fractions in the *Calr* ^−/−^ cells was found in fraction 7 and 8 (Fig. 7B). These results demonstrated that the ER distribution of unesterified cholesterol, which regulates the processing of nSREBP, was altered by the reduction of intracellular Ca^2+^.

## Discussion

The mechanism controlling cellular cholesterol homeostasis was elucidated by the Brown and Goldstein laboratory^14, 16, 31–33^. This elegant machinery is comprised of several cellular components that enable the cell to sense the concentration of intracellular cholesterol and maintain its concentration within a narrow physiological range^32^. According to the current state of knowledge a small portion of unesterified cholesterol is found in the ER membrane where it controls the processing of ER membrane-bound SREBP to the active form of the transcription factor that is responsible for the expression of genes involved in lipid metabolism^14, 17, 34^. The SCAP/SREBP complex is retained in the ER when there is an excess of cholesterol in the ER^17^ thereby preventing the processing of the SREBP precursor by S1- and S2-proteases that reside in the Golgi. This ER cholesterol store is fed by two of three distinct plasma membrane cholesterol pools. The larger of these two pools is easily accessible by the bacterial protein Perfringolysin O (PFO) variant, that binds unesterified cholesterol in membranes, and the smaller pool becomes PFO accessible only after treatment of the plasma membrane with sphingomyelinase^35^. The remaining pool is termed the “essential” plasma membrane cholesterol since depletion of this pool causes cell death^36^. The cellular components responsible for the transport of cholesterol from the plasma membrane to the ER as well as the itinerary of the transport vesicles are not yet determined, although it is well accepted that the concentration of cholesterol that ends up in the ER is what controls the fate of the SCAP/SREBP complex.

**Figure 8:**
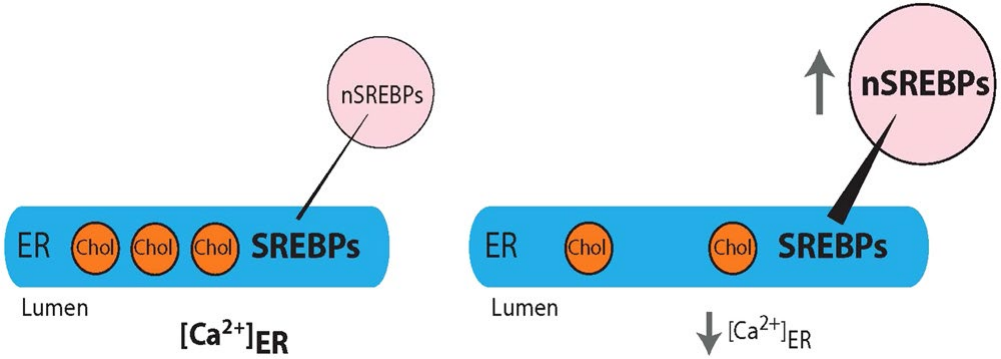
A schematic representation depicting the role of Ca^2+^ on intracellular cholesterol distribution. The model shows cross-talk between endoplasmic reticulum (*ER*) Ca^2+^ and intracellular cholesterol distribution. The majority of unesterified cholesterol (*Chol*) in the cell is localized in the plasma membrane (PM) and the remainder is found in ER membranes where it regulates the processing of SREBP. Under cholesterol replete conditions (*left*) SREBP processing is limited. Reducing ER luminal Ca^2+^ (*right*) enriches a distinct intracellular pool of cholesterol. The decrease in the ER membrane cholesterol enhances SREBP processing and increases the abundance of SREBP in the nucleus.

In this study, we provide evidence illustrating a critical role of ER Ca^2+^ in the lipid homeostasis in eukaryotic cells. We showed that the enhanced nSREBP activity in calreticulin-deficient cells, which have reduced ER Ca^2+^ ^10^, can be normalized to a level comparable to that in wild-type cells simply by expressing the Ca^2+^ binding domain of calreticulin. Conversely, we showed that depletion of ER Ca^2+^ in wild-type cells, independently of calreticulin, caused the enhancement of nSREBP activity in a concentration-dependent manner. The effect of ER Ca^2+^ on enhanced nSREBP activity was not dependent on the concentration of intracellular unesterified cholesterol, ER stress, nor the processing by S1P and S2P proteases. Importantly, our analysis indicated that the cholesterol sensing mechanism of the SREBP pathway remained fully functional in cells with reduced ER Ca^2+^. Surprisingly however, through direct staining and subcellular fractionation, we found that cells with reduced ER Ca^2+^ showed modified intracellular distribution of unesterified cholesterol. Therefore, we propose that reduced ER Ca^2+^ does not impact on the total amount of intracellular unesterified cholesterol but shifts the distribution of cholesterol within the ER to a pool that is not directly accessible to the components of the cholesterol sensing mechanism (Fig. 8). ER Ca^2+^ status may be a previously unrecognized determinant of the basal sensitivity of sterol sensing mechanism inherent to the SREBP processing pathway.

## Experimental Procedures

### Ethics

All methods were carried out in accordance with relevant guidelines and regulations and approved by the the Biosafety Officers at the Department of Environment, Health and Safety at the University of Alberta. All animal experiments were carried out according to the University of Alberta Animal Policy and Welfare Committee and the Canadian Council on Animal Care Guidelines. The approval for use of animals in research was granted by the Animal Care and Use Committee for Health Sciences, a University of Alberta ethics review committee. The protocol was approved by the Committee (Permit AUP297). All animal experimentation was carried out working closely with University of Alberta animal facility staff and veterinarian.

### Cell culture

Wild-type and *Calr* ^−/−^ mouse embryonic fibroblasts (referred to as wild-type and *Calr* ^−/−^ cells, respectively) were previously described^10^. *Calr* ^−/−^ cells stably expressing full length calreticulin, or portions of calreticulin representing the protein chaperone region (N+ P domain) or Ca^2+^ binding domain (P+ C domain) of calreticulin were previously described^10^. Cells were cultured in Dulbecco’s Modified Eagle’s Medium (DMEM) (Sigma-Aldrich) with 10% fetal bovine serum (FBS) (Sigma-Aldrich). Some experiments used lipid-free media which is DMEM supplemented with 10% lipoprotein deficient fetal bovine serum (Sigma-Aldrich #S5394).

Cells were transfected with plasmid DNA (0.25 µg) using DharmaFECT Duo transfection reagent (ThermoFisher Scientific) into 48-well plates for 24–48 h. Reporter activity (luciferase) was measured using a Dual-Luciferase® Reporter Assay System (Promega), which measures Firefly luciferase activity under the control of a test promoter normalized to Renilla luciferase activity under the control of the reference (CMV) promoter. The human SREBP-2 promoter containing a canonical and functional sterol response element (SRE)^36, 37^ upstream of the Firefly luciferase structural gene sequence was used as the SREBP activity reporter (referred to as SRE luciferase reporter), was a kind gift from Dr. Zlokovic’s lab^38^. To confirm the functionality of the SRE in the human SREBP2 gene promoter, the SRE sequence (5′-ATCACCCCAC-3′) was converted to 5′-ATTACCACGC-3′ to create a mutated SRE luciferase reporter plasmid (referred to as mutSRE luciferase reporter) using the following DNA primers: the forward primer: 5′-CGATGACGCGCCATTACCACGC-3′ and the reverse primer: 5′-CGAAGCGGTGCGCGTGGTAATGGCGCGTCATCG-3′. Nuclear SREBP-2 (nSREBP) activity reported in this study was assessed using a Dual Luciferase® Reporter System, cells were co-transfected with the SRE luciferase reporter vector [reporting nuclear SREBP (nSREBP) activity]^38^ and the pGL4.75 plasmid at a 40:1 ratio, followed by treatment and luciferase assay. SRE-mediated responses to the cholesterol status was confirmed by co-transfection of cells with the control SRE luciferase reporter plasmid or the mutSRE luciferase plasmid with the pGL4.75 plasmid (40:1 ratio), treated with normal media or lipid-free media without or with cholesterol (0.25 µg/ml) followed by luciferase assay (Supplementary Fig. S7). Transfection efficiency for each cell line used in this study was determined by the fluorescence-activated cell sorting analysis of cells transfected with RFP expression vector. The transfection efficiency reported as a percentage were as follows: 44.56 ± 0.58% (wild-type cells), 63.11 ± 4.91% (*Calr* ^−/−^ cells), 59.74 ± 1.91% (HeLa cells) and 50.50 ± 0.24% (HEK293T cells). The Cholesterol Biosynthesis (SREBP) Pathway biomarkers were from SwitchGear Genomics. For luciferase activity assays, cells were harvested with Passive Lysis Buffer (50% glycerol, 2.5% trans-1,2-diaminocyclohexane-N,N,N,‘N’-tetraacetic acid monohydrate and 0.5% N,N-Bis(3-D-gluconamideopropyl)cholamide) (Promega) and the bioluminescence of Firefly and Renilla luciferases were measured using AutoLumat Plus LB 953 luminometer (Berthold Technologies).

For assessment of sterol sensitivity and SCAP transport, cells were transfected with luciferase reporter plasmids and cultured for 24 h in either DMEM with 10% FBS (complete media) or DMEM with 10% lipid-free FBS (Sigma-Aldrich #S5394) (lipid-free media) without or with differing concentrations of methyl-β-cyclodextrin encapsulated cholesterol (Sigma-Aldrich #C4951). To deplete ER Ca^2+^, cells were treated with 0.5 µM thapsigargin. Extracellular Ca^2+^ concentration was controlled by addition of EGTA to the cell culture media. The EGTA calculator MaxChelator (maxchelator.stanford.edu) was used to calculate the concentration of EGTA required ensuring specific free Ca^2+^ concentrations. Total free Ca^2+^ in growth media was determined to be 2.17 mM (1.8 mM from DMEM and 0.37 mM from FBS). To achieve the following extracellular Ca^2+^ concentrations, 500 µM, 300 µM, 150 µM and 100 µM, the following EGTA concentrations were added 1.67 mM, 1.87 mM, 2.02 mM and 2.08 mM, respectively. Cells were incubated in low Ca^2+^ media for 24 h. For assessment of ER to Golgi transport cells were treated with 1 µg/ml Brefeldin A (Sigma-Aldrich) for 16 h. For S1P or calreticulin silencing, cells were transfected with scrambled siRNA or siRNA specific for S1P or calreticulin (Santa Cruz Biotechnology) using DharmaFECT transfection reagent (Thermo Fisher Scientific). For induction of ER stress, cells were treated with 5 ng/ml tunicamycin (Sigma-Aldrich) for 24 h. The XBP1 splicing reporter assay was carried out as previously described^28^. For Ca^2+^ measurements wild-type, *Calr* ^−/−^ cells or *Calr* ^−/−^ cells expressing calreticulin domains were loaded with Fura-2/AM and fluorescence was measured as described previously^39^. Ca^2+^ release from ER stores was induced by treatment with 1 µM thapsigargin.

### *C. elegans* analysis

For Sudan Black B staining larvae and young adults were fixed in a buffer containing 80 mM KCl, 20 mM NaCl, 7 mM Na_2_ EGTA, 15 mM PIPES, pH 7.4, 0.5 mM spermidine, 0.2 mM spermine, 0.1% β-mercaptoethanol, 2% paraformaldehyde, and subjected to three freeze-thaw cycles. Worms were then washed with 70% ethanol, and stained in a saturated solution of Sudan Black B in 70% ethanol. DIC images were captured using Zeiss Axio Imager microscope. CE548 worms expressing *epEx141*[*sbp*-*1*::*GFP*::*SBP*-*1*] in *sbp*-*1*(*ep79*) background was crossed with *crt*-*1*(*jh101*) to visualize nuclear localization of GFP:SBP-1^40, 41^. Fluorescent images were captured, and the ratio of fluorescence intensity in the nucleus and cytoplasm immediately outside the nucleus was analyzed using ImageJ.

### Immunoprecipitation and immunoblot analyses

Cells plated in 10 cm plates were harvested at 80–90% confluency with 500 µl Lysis Buffer (50 mM Hepes, pH 7.4, 200 mM NaCl, 2% CHAPS) with protease inhibitors (0.5 mM phenylmethylsulfonyl fluoride, 0.5 mM benzamidine, 0.05 µg/mL Na-tosyl-Lys-chloromethylketone, 0.05 µg/ml 4-amidoinophenyl-methanesulfonyl fluoride hydrochloride monohydrate, 0.05 µg/ml (2S,3S)-3-(N-f3dcarbamoyl) oxirane-2-carboxylic acid (E-64), 0.025 µg/ml leupeptin and 0.01 µg/ml pepstatin) for 30 min on ice. Cell lysates were pelleted by centrifugation at 11,200 × g for 15 min. The supernatant was diluted with the addition of a 2/3 volume of Lysis Buffer and precleared with 1/15^th^ volume of 10% Protein A Sepharose CL-4B bead (GE Healthcare Life Sciences) suspended in HBS (50 mM Hepes, pH 7.4, 200 mM NaCl) for 30 min at 4 °C then pelleted for 10 sec. The supernatant was collected and incubated overnight at 4 °C with 2–3 µl of specific antibodies. The sample was incubated with 100 µl of 10% Protein A beads for 4 h at 4 °C, then washed 5 times with HBS in the presence of 1% CHAPS, 2 times with HBS, and re-suspended in 30 µl SDS-PAGE sample buffer (60 mM Tris-HCl, pH 6.8, 1% SDS, 10% glycerol and 3% β-mercaptoethanol and 0.01% bromophenol blue) followed by immunoblot analysis.

Immunoblot analysis was carried out as previously described^28^. Antibodies used were mouse anti-SREBP-1 (1:200; Santa Cruz Biotechnology sc-13551), rabbit anti-SREBP-2 (1:500; Abcam ab30682), rabbit anti-INSIG1 (1:500; Cedarlane 22115-1-AP), rabbit anti-SCAP (1:500; Cedarlane 12266-1-AP), goat anti-calreticulin (1:500), rabbit anti-calnexin (1:2000; Stressgen Bioreagents ADI-SPA-860), rabbit anit-BiP (1:2000, Abcam ab21695), rabbit anti-GAPDH (1:2000; Thermo Fisher Scientific), mouse anti-ATF6 (1:500; IMGENEX IMG-273) and mouse anti-γ-tubulin (1:1000; ThermoFisher Scientific MA1-850). Secondary antibodies used were HRP conjugated: rabbit anti-mouse, goat anti-rabbit or rabbit anti-goat (1:2000; Jackson ImmunoResearch). Immunoblot images were scanned and quantified by densitometry using Image Studio Lite Ver 5.0 (Li-Cor).

### Q-PCR analysis

Total RNA was isolated from cells using the RNeasy Mini Kit (QIAGEN) and first strand cDNA synthesis was performed with iScript Reverse Transcription Supermix for RT-qPCR (BIO-RAD). For the PCR reaction, we used PerfeCTa SYBR Green FastMix (Quanta BioSciences) and quantification was performed on Rotor-Gene Q (QIAGEN). The following primers were used: SREBP-1 Forward: 5′-GCGGCTGTTGTCTACCATAA-3′; SREBP-1 Reverse: 5′-CTGGGCTAGATTCCACCTTTC-3′; SREBP-2 Forward: 5′-GAGGCGGACAACACACAATA-3′; SREBP-2 Reverse: 5′-CGGCTCAGAGTCAATGGAATA-3′; LDLR Forward: 5′-TGCATTTTCCGTCTCTACACT-3′; LDLR Reverse: 5′-CAACGCAGAAGCTAAGGATGA-3′; HMGR Forward: 5′-ACTGACATGCAGCCGAAG-3′; HMGR Reverse: 5′-CACATTCACTCTTGACGCTCT-3′; SQLE Forward: 5′-GCTCCTGTTAATGTCGTTTCTG-3′; SQLE Reverse: 5′-TCTCTGCTTTGCCTCTTATTGG-3′; LSS Forward: 5′-GCAGAGAGATGTGTGATATGTGA-3′; LSS Reverse: 5′-AGGCTGAGGATGGACACT-3′; IDI1 Forward: 5′-CATCAGATTGGGCCTTGTAGT-3′; IDI1 Reverse: 5′-ATTGGTGTGAAGCGAGCA-3′; REPIN1 Forward: 5′-CTGTTCCAGCATCGGTTCT-3′; REPIN1 Reverse: 5′-GCAGTTGTGAACTCGAACCT-3′.

### Lipid staining and cholesterol localization analyses

Cells were cultured on 25 mm cover slips and the indicated treatments were applied. For neutral lipid staining, cells were washed 3 times with PBS, fixed for 30 min with 3.7% formaldehyde (in PBS), washed 3 times with PBS, incubated for 1 h at 37 °C with stain prepared by diluting the BODIPY 505/515 stock (1 mg/ml in 100% ethanol) 1/1000 in PBS. The cover slips were washed 3 times with PBS before visualization.

For unesterified cholesterol staining, cells were incubated for 30 min with 60 µl/ml of NucRed Live 647 (Molecular Probes - Life Technologies), washed 3 times with PBS, fixed for 1 h in 3.7% formaldehyde (in PBS), washed 3 times with PBS, incubated for 10 min with 1.5 mg/ml glycine (in PBS), incubated with 0.05 mg/ml filipin (Sigma-Aldrich) for 2 h and washed 3 times with PBS before visualization.

For ER and unesterified cholesterol co-localization, cells were transfected with an ER-RFP expressing plasmid (2.5 µg), using the NEON transfection system (Thermo Fisher Scientific) prior to filipin staining. For ER marker and SREBP-2 co-localization, cells were transfected with an ER-RFP expressing plasmid (2.5 µg), washed 3 times with PBS, fixed for 1 h in 3.7% formaldehyde (in PBS) and washed 3 times with PBS. Cells were permeabilized with 0.3 M glycine in PBS with 0.1% Tween, washed 3 times with PBS, incubated with rabbit anti-SREBP-2 (1 µg/ml) overnight at 4 °C, washed 3 times with PBS, incubated with goat anti-rabbit Alexa Fluor® 305 (1:1000 dilution) for 2 h and washed 3 times with PBS before visualization. Staining was visualized using a Leica TCS SP5 microscope. For BODIPY FL-labelled low density lipoprotein (LDL) uptake, cells were serum starved for 24 h followed by incubation with BODIPY FL-labelled LDL (10 µg/ml) in serum free media for 0, 10, 15, 30 and 60 min. Cells were washed 3 times with PBS, fixed for 1 h in 3.7% formaldehyde (in PBS) and washed 3 times with PBS before visualization. For visualization of BODIPY 505/515 staining, the Argon laser was used with excitation at 488 nm and emission from 505–515 nm. For BODIPY FL-LDL visualization, the Argon laser was used with excitation at 514 nm and emission from 520–580 nm. For NucRed, the Red HeNe laser was used with excitation at 633 nm and emission peak at 686 nm. For filipin staining, the UV laser was used with excitation at 405 nm and emission peak at 460 nm. For Alexa Fluor® 305, the UV laser was used with excitation at 405 nm and emission peak at 430 nm. For ER-RFP fluorescence, the Red HeNe laser was used with excitation at 514 nm and emission peak at 584 nm.

Overlap of filipin and ER-RFP signals was analyzed using ImageJ (https://imagej.nih.gov/ij/download.html). A straight line was drawn across the middle of each cell and identified as a region of interest (ROI). The signal intensity of each channel (blue for filipin and red for RE-RFP) for each cell was obtained using the corresponding ROI and the values were plotted along the same X axis coordinates to identify regions of overlap.

### Lipid biosynthesis and measurement

Wild-type and *Calr* ^−/−^ cells were cultured in DMEM (Sigma) with 10% FBS (control) or 10% lipid-free serum in 6 cm dishes for 24 h. Cells were then washed once with warm lipid-free DMEM and incubated for 4 h in 2 ml of lipid-free DMEM containing 50 µM acetate and 10 µCi [^3^H] acetate (specific activity 18.5 GBq/mmol). Following the incubation, cells were washed 2 times with PBS, harvested in 2 ml PBS and lysed by sonication. For normalization, 500 µl of each lysate were taken for DNA concentration determination. Total lipids from 1 ml of lysate were extracted using the Folch method (chloroform:methanol, 2:1), dried under nitrogen, solubilized in 100 µl of chloroform and separated by thin-layer chromatography (TLC) using heptane/isopropyl ether/acetic acid (60/40/4 by volume) as the mobile phase. TLC plates were exposed to iodine vapor for visualization of lipid classes then the lipid bands were scraped from the TLC plate and the radioactivity associated with each lipid class was determined by liquid scintillation spectrometry. The sample radioactivity was normalized to the DNA concentration of the starting sample. For the measurement of total triglycerides, cholesterol esters and unesterified cholesterol, wild type and *Calr* ^−/−^ cells were homogenized in PBS and passed through an 18 micron clearance ball bearing homogenizer 25 times. Extraction of lipids from the homogenate was performed using the Folch method as previously described^42^ and the lipid classes were analyzed using high-performance liquid chromatography (Lipodomics, University of Alberta). The concentration of total triacylglycerols, and total and unesterified cholesterol in cell homogenates was measured using commercially available colorimetric assays (Stanbio Laboratory, Boerne, TX).

### Subcellular fractionation and gas chromatography analysis of cholesterol content

Wild-type and *Calr* ^−/−^ cells were grown in 150 mm dishes to 90% confluency. Four dishes were used per each gradient separation. Gradients were prepared one day prior to cell harvest. Optiprep (60% iodixanol in water) (Sigma-Aldrich) was used and diluted to a working solution of 50% iodixanol in Diluent buffer (0.25 M sucrose and 60 mM Tris, pH 7.4). 6%, 9%, 12%, 15%, 18%, 21%, 24% and 27% dilutions of iodixanol was prepared with the working solution in homogenization buffer (0.25 M sucrose and 10 mM Tris, pH 7.4) and 500 μl of each dilution was sequentially layered in polyallomer (13 × 51 mm) centrifuge tubes. The formed gradients were stored at 4 °C overnight. Cells were washed 2 times with PBS, and harvested on ice in 800 μl homogenization buffer. The homogenate was passed through an 18 micron clearance ball bearing homogenizer 30 times and centrifuged at 800 xg for 10 minutes. One hundred μl of homogenate was kept for total lipid analysis and 800 μl of the homogenate was layered on top of the iodixanol gradient. The gradients were centrifuged at 39,000 rpm for 6 h and twelve fractions (400 μl per fraction) were collected from the top of gradients into 1.5 ml Eppendorf tubes. One hundred μl of each fraction were taken for protein isolation and the remaining 1 ml of each fraction were taken for lipid extraction for gas chromatography analysis.

Proteins were precipitated by addition of 300 μl of 100% acetone to each fraction and incubated at 4 °C overnight. The samples were then centrifuged at 13,200 rpm for 10 min at 4 °C. The pellets were washed with 100% ethanol and the re-centrifuged at 13,200 rpm for 10 min at 4 °C. The resulting pellets were dissolved in 100 μl SDS-PAGE sample buffer and then processed for immunoblot analysis.

For lipid extraction, individual fractions were mixed with 2 ml of solution containing 17.5 mM Tris, pH 7.3, 10 mM CaCl_2_, 2 units of phospholipase C from *Clostridium welchii* (Sigma) and 2 ml diethyl ether. The samples were mixed and incubated at 30 °C for 2 h with constant mixing. One ml of tridecanoin (2 μg/ml in chloroform) and 6 ml of chloroform:methanol (2:1) was added followed by centrifugation at 2,500 rpm for 10 min. The lower phase was removed and passed through a Pasteur pipette containing anhydrous Na_2_SO_4_ into a smaller glass tube. The resulting mixture was dried under nitrogen. The remaining residue was dissolved in 100 µl Sylon BFT (Supelco),incubated at room temperature for 1 h and analyzed by gas chromatography (Agilent Technologies, 6890 Series equipped with a flame ionization detector; Palo Alto, CA). Samples were injected onto an Agilent high performance capillary column (HP-5, 15 m × 0.32 mm × 0.25 µm). The oven temperature was raised from 170 to 290 °C at 20 °C/min and then to 340 °C at 10 °C/min, with helium as a carrier gas (87 cm/s) with a constant flow rate of 4.5 ml/min^43^.

### Statistical analysis

All statistical analyses were performed using the GraphPad Software. Means were compared using the Student’s t-test or ANOVA where appropriate. Significant differences were attributed to *p*-values < 0.05.

## Electronic supplementary material

Supplemental Figures
