# Supplementary material for: Loss of Calreticulin Uncovers a Critical Role for Calcium in Regulating Cellular Lipid Homeostasis
Source: Sci Rep. 2017 Jul 19;7:5941. doi: 10.1038/s41598-017-05734-x (PMC5517566; doi:10.1038/s41598-017-05734-x)
Supplement: Supplementary file 1 — Supplemental Figures [file 41598_2017_5734_MOESM1_ESM.pdf]

SUPPLEMENTARY MATERIAL

Loss of Calreticulin Uncovers a Critical Role for Calcium in Regulating Cellular Lipid Homeostasis

Wen-An Wang[1], Wen-Xin Liu[1], Serpen Durnaoglu[2], Sun-Kyung Lee[2], Jihong Lian[3], Richard Lehner[3], Joohong Ahnn[2], Luis B. Agellon[4] and Marek Michalak[1]

From the [1]Department of Biochemistry, University of Alberta, Edmonton, Alberta, Canada T6G 2H7; [2]Department of Life Sciences, Research Institute for Natural Sciences, BK21 Plus Life Science for BDR Team, Research Institute of Natural Science, Hanyang University, Seoul 133-791, South Korea; [3]Department of Cell Biology, University of Alberta, Edmonton, Alberta, T6G 2H7, Canada and [4]School of Dietetics and Human Nutrition, McGill University, Ste. Anne de Bellevue, Quebec, H9X 3V9, Canada

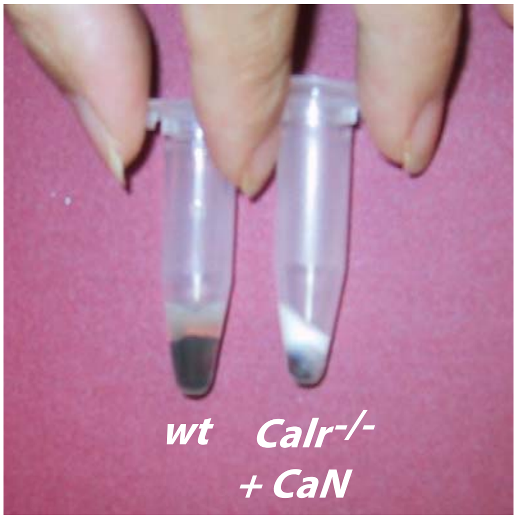

Supplementary Figure S1. Serum from wild-type (*wt*) and calreticulin-deficient (*Calr*$^{-/-}$) mouse rescued by cardiac-specific expression of the activated calcineurin (*Calr*$^{-/-}$+CaN)[12].

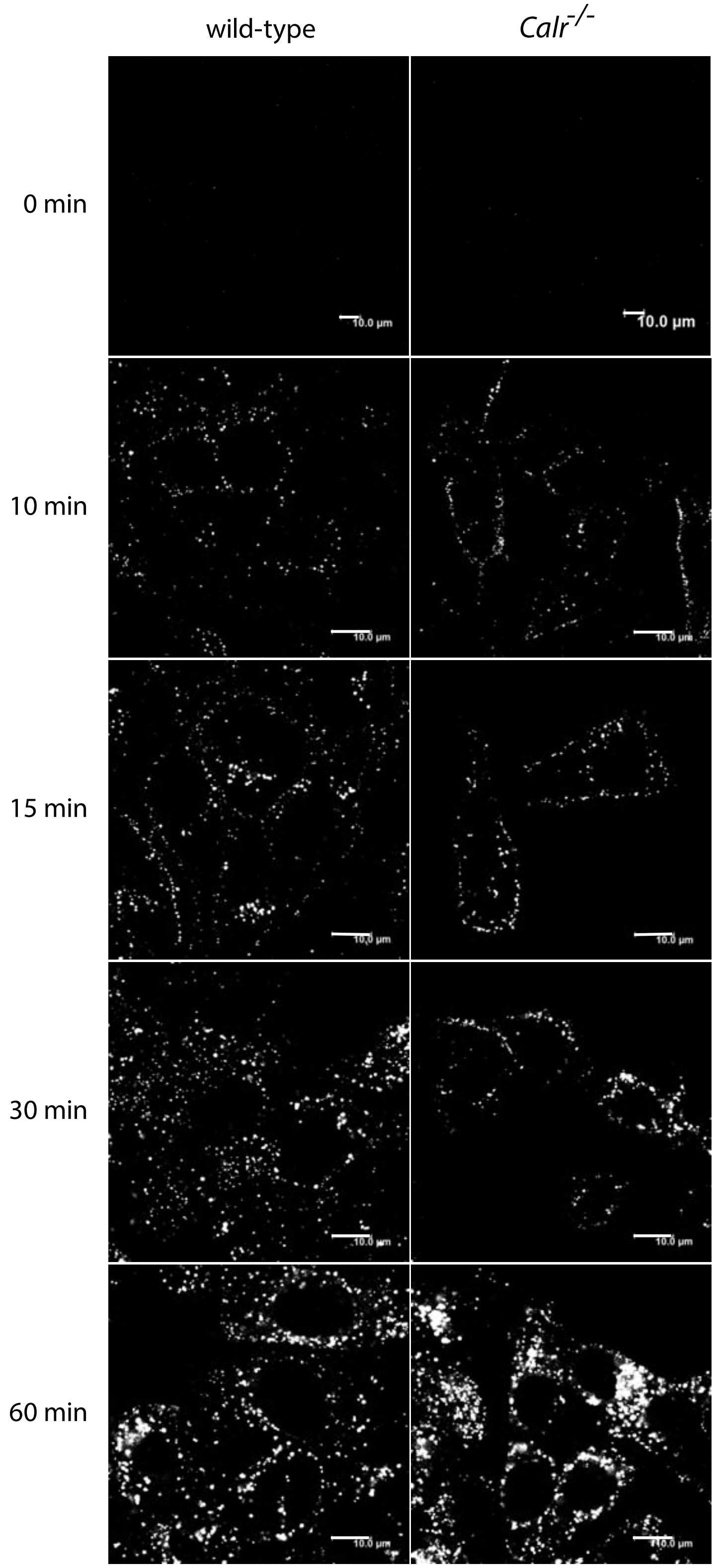

Supplementary Figure S2. Uptake of fluorescent LDL in wild-type and *Calr*$^{-/-}$ cells. The time of incubation with LDL fluorescent complex with BODIPY as indicate in the Figure.

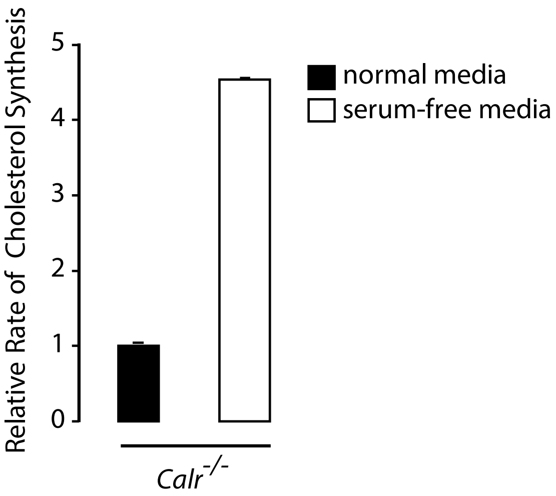

Supplementary Figure S3. Rate of cholesterol synthesis in *Calr*$^{-/-}$ cells cultured with normal and serum-free media.

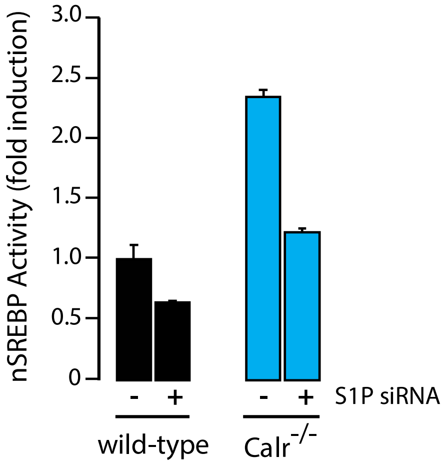

Supplementary Figure S4. Wild-type and *Calr*$^{-/-}$ cells were transfected with siRNA specific for S1P followed by nSREBP activity assay.

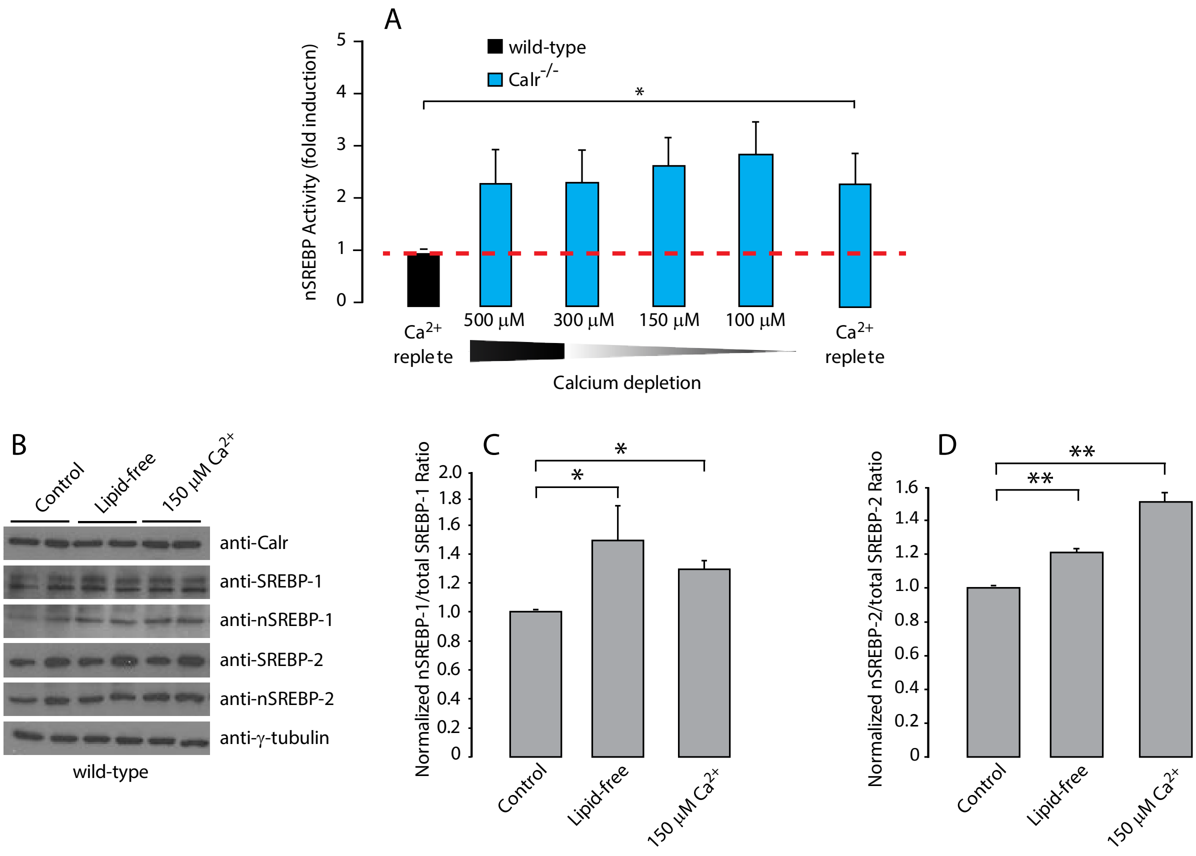

Supplementary Figure S5. Ca^{2+} effects on SREBP processing in *Calr$^{-/-}$* cells. A. nSREBP activity in *Calr$^{-/-}$* cells exposed to decreasing extracellular Ca^{2+} concentration as indicated in the Figure. Ca^{2+} replete represents 2.17 mM extracellular Ca^{2+} concentration. *Indicates statistically significant differences, wild-type vs. *Calr$^{-/-}$* cells in control conditions *p*-value=0.0235. Representative of 7 biological replicates. **B.** Representative immunoblot analysis of SREBP-1, nSREBP-1, SREBP-2 and nSREBP-2 protein in wild-type cells grown in cholesterol-free media or treated with 150 µM extracellular Ca^{2+}. Anti-γ-tubulin antibodies were used as a loading control. Representative of 3 biological replicates. **C, D.** Quantitative analysis of Immunoblots, ratio of nuclear to total SREBP-1 (**C**) and total SREBP-2 (**D**). *Indicates statistically significant differences, in **C**, for SREBP-1, control vs. cholesterol-free *p*-value=0.0432 and control vs. 150 µM Ca^{2+}, *p*-value=0.0146. **Indicates statistically significant differences, in **D**, for SREBP-2, control vs. cholesterol free, *p*-value=0.0012; control vs. 150 µM Ca^{2+}, *p*-value=0.0013. Representative of 3 biological replicates. The images of (**B**) shown are cropped. The full-length gel/blots are shown in Fig. S17.

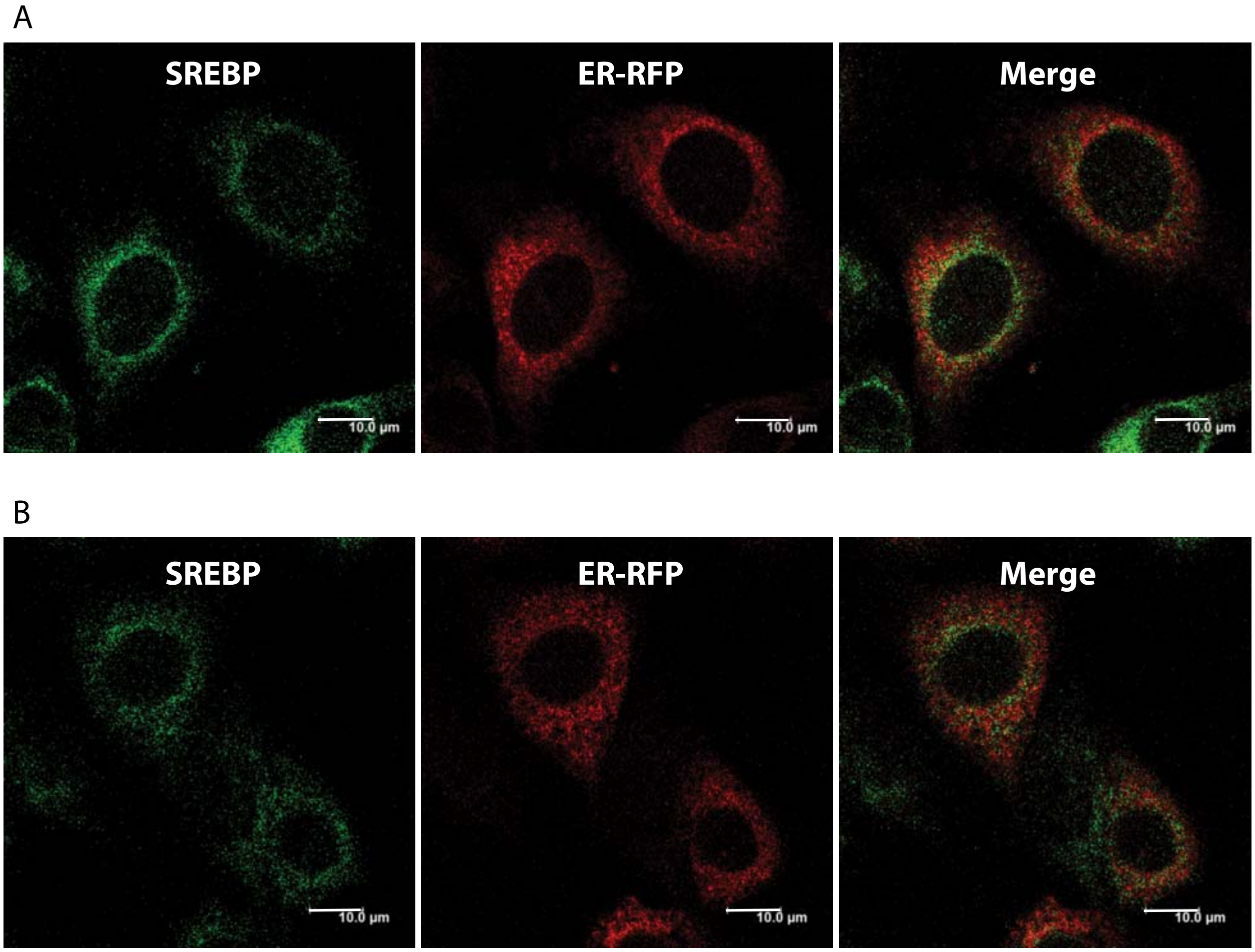

Supplementary Figure S6. Cellular distribution of SREBP-2 in wild-type and *Calr*$^{-/-}$ cells. Wild-type cells (**A**) and *Calr*$^{-/-}$ cells (**B**) expressing ER-targeted red fluorescent protein (ER-RFP) were stained with anti-SREBP antibodies. Wild-type cells Pearson's coefficient=0.231±0.035 (n=15); *Calr*$^{-/-}$ cells Pearson's coefficient=0.291±0.038 (n=15). Graphic representation of overlap SREBP and ER-RFP signals from representative cells is presented in the Figure. The arrows indicate the direction of the scan represented in the graph.

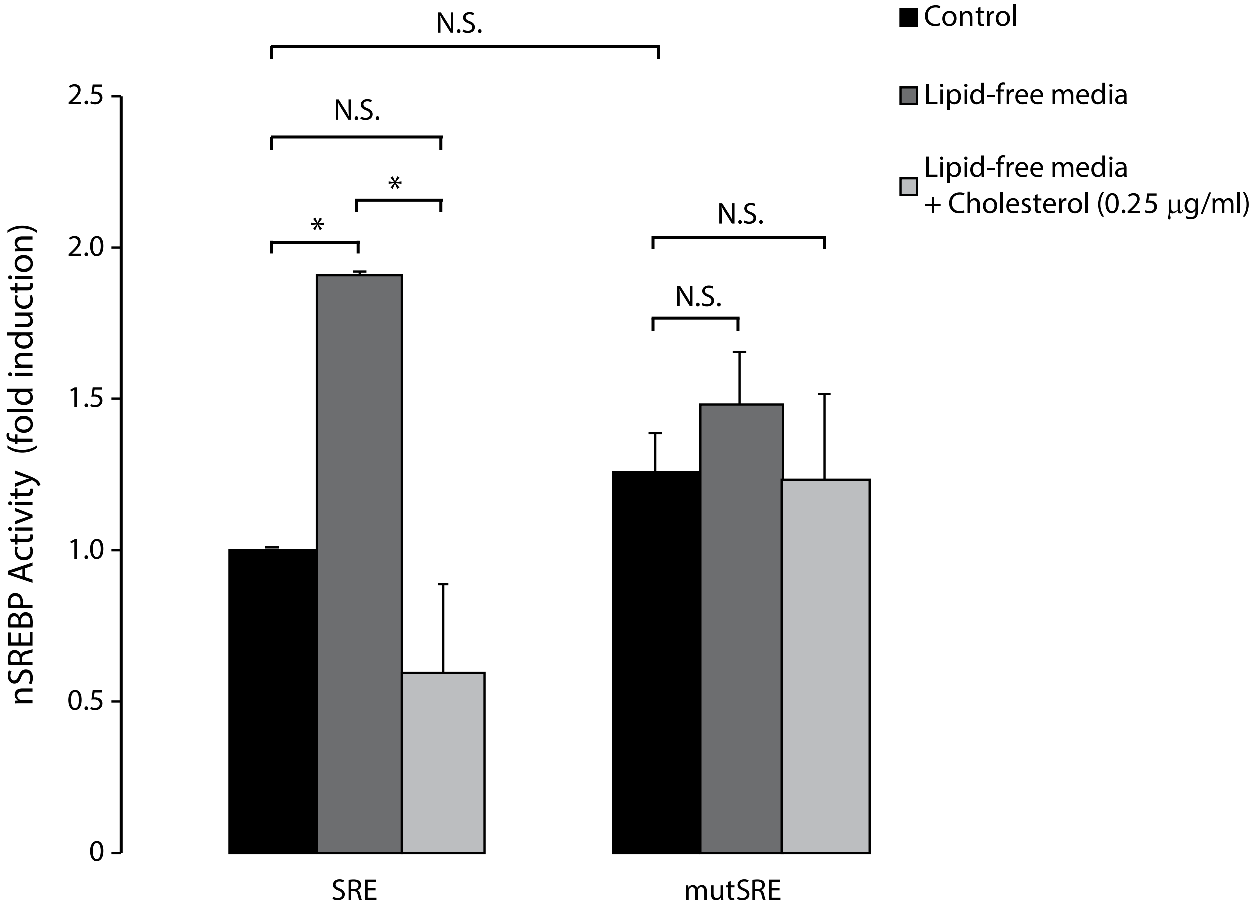

Supplementary Figure S7. Mutational analysis of the SRE element in HeLa cells. nSREBP activity in HeLa cells transfected with the SRE luciferase reporter plasmid or the mutated SRE (mutSRE) luciferase reporter plasmid treated with normal media, lipid-free media and lipid-free media plus cholesterol (0.25 µg/ml). SRE nucleotide sequence was mutated from ATCACCCCAC to ATTACCACGC. *Indicates statistically significant differences: HeLa cells with the SRE luciferase reporter in normal vs. lipid-free media, p-value<0.05 (ANOVA); HeLa cells with the SRE luciferase reporter in lipid-free media vs. lipid-free media plus cholesterol (0.25 µg/ml), p-value<0.05 (ANOVA). NS, not significant. Representative of 3 trials with 3 replicates.

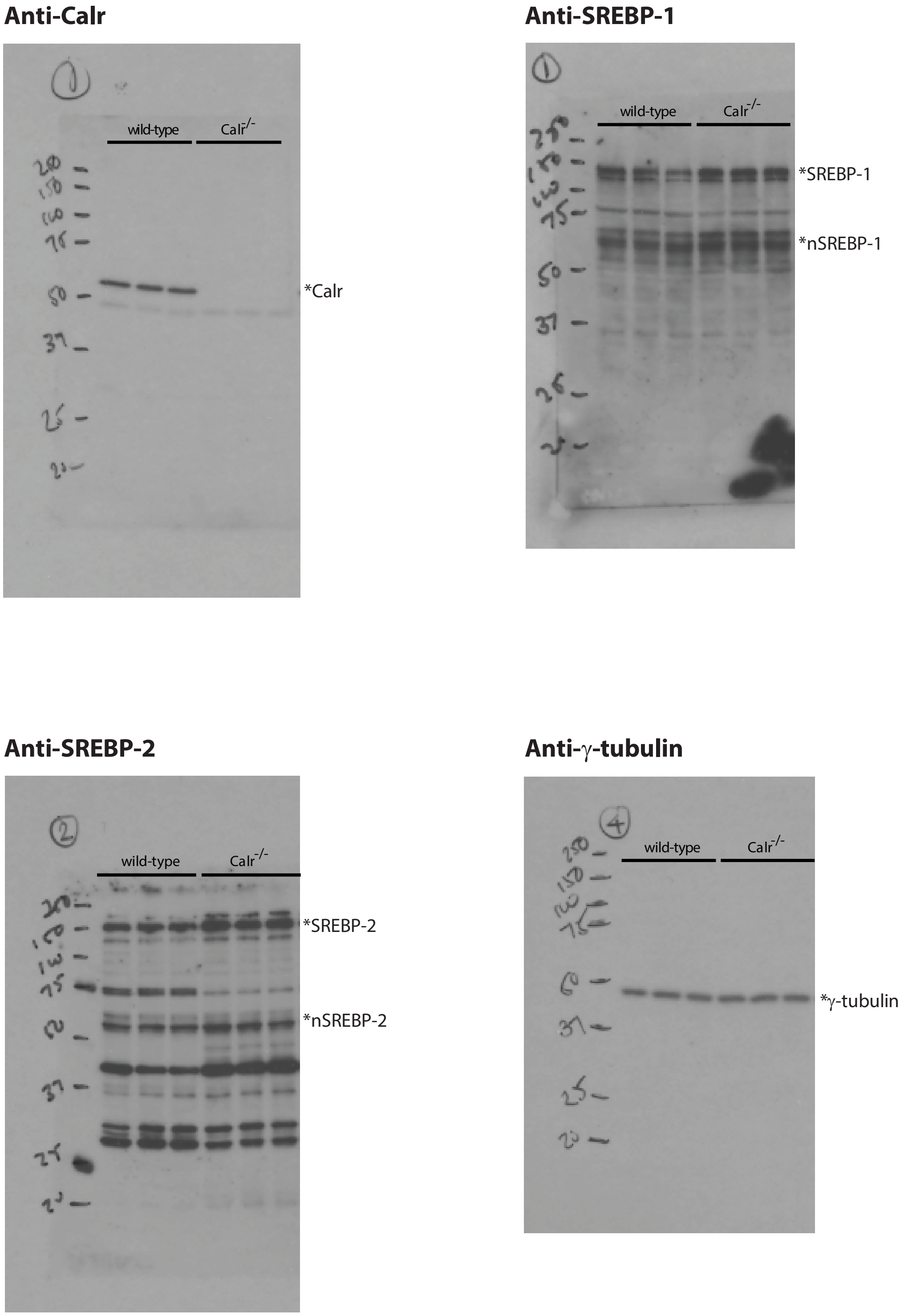

Figure S8. The full-length blots for Figure 2B

Anti-Calr

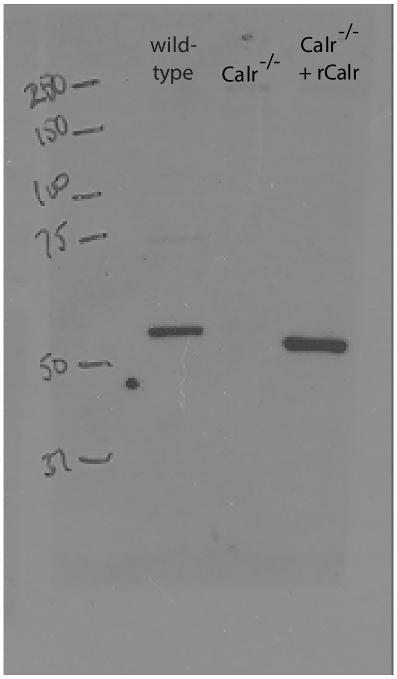

Figure S9. The full-length blots for Figure 2F

Anti-Calr

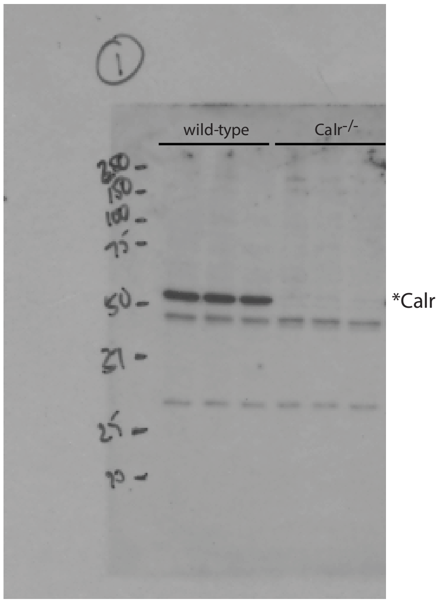

Anti-SCAP

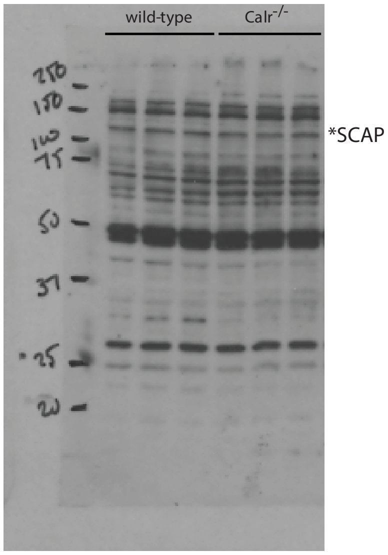

Anti-INSIG

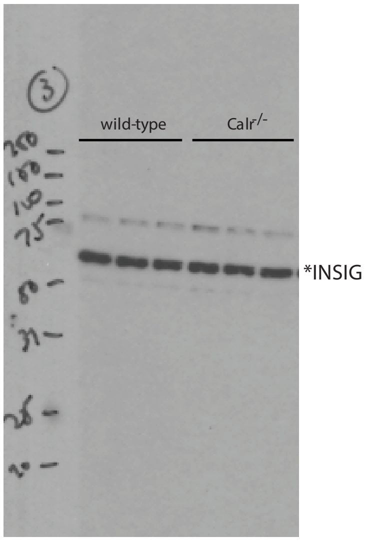

Anti-γ-tubulin

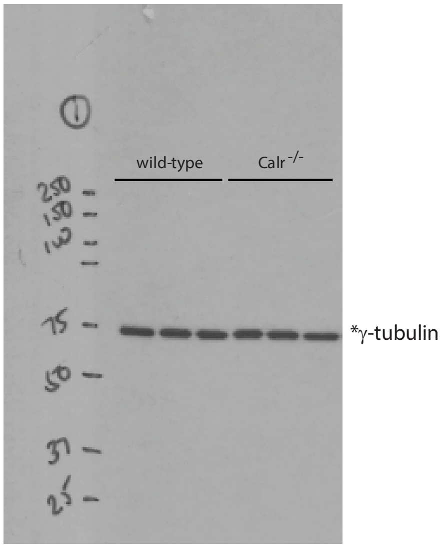

Figure S10. The full-length blots for Figure 3B

Anti-INSIG

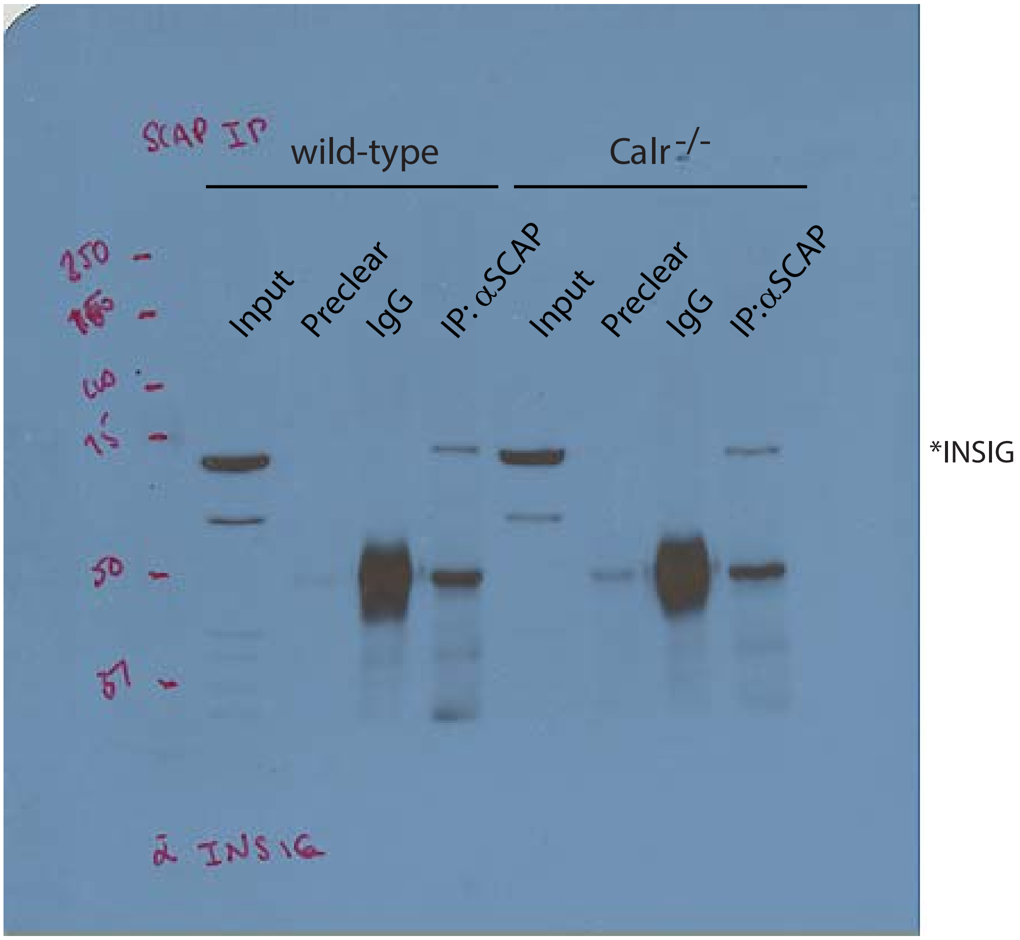

Figure S11. The full-length blot for Figure 3C

Anti-SCAP

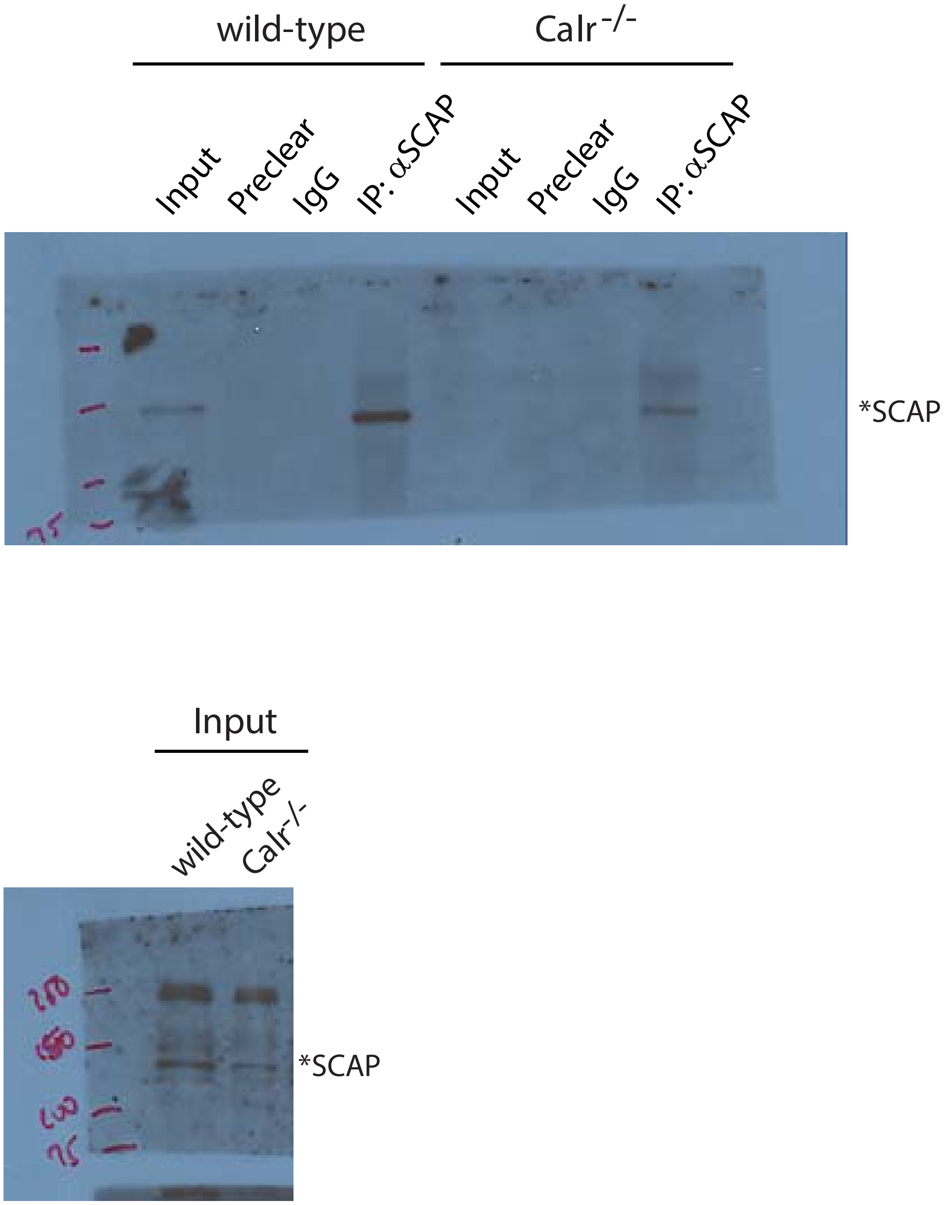

Figure S12. The full-length blots for Figure 3D

Anti-SREBP-2

wild-type

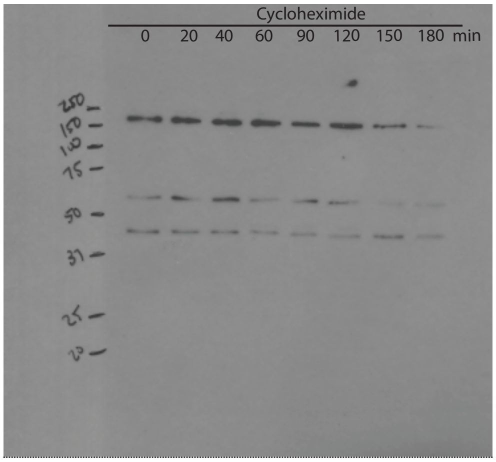

*nSREBP-2

Calr$^{-/-}$

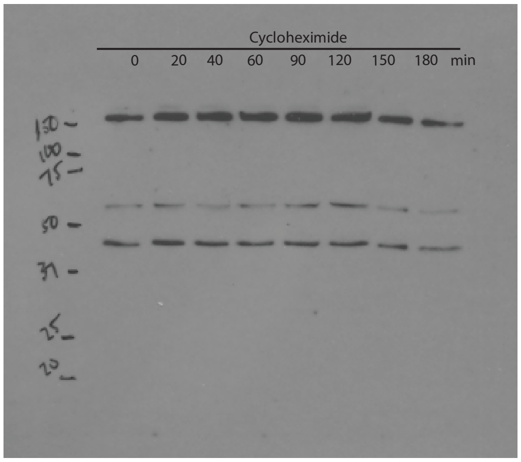

*nSREBP-2

Anti-γ-tubulin

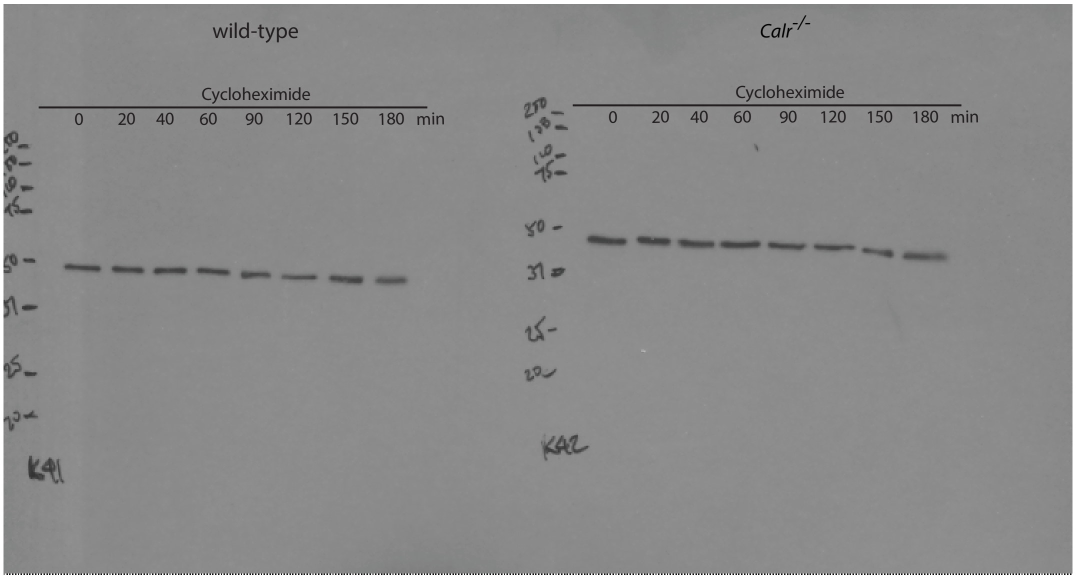

*γ-tubulin

Figure S13. The full-length blots for Figure 3F

Anti-ATF6

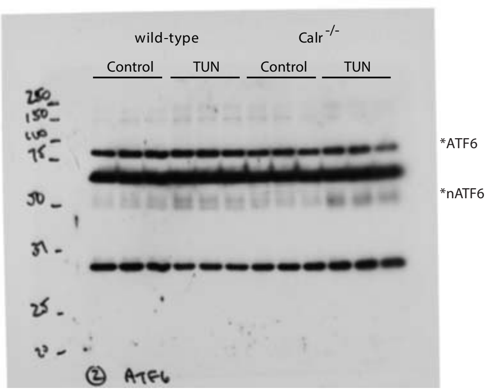

Anti-Calr

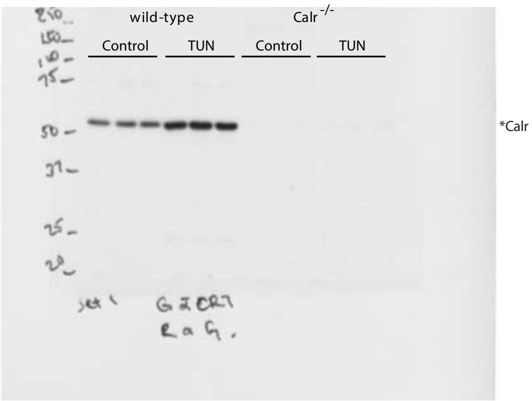

Anti-γ-tubulin

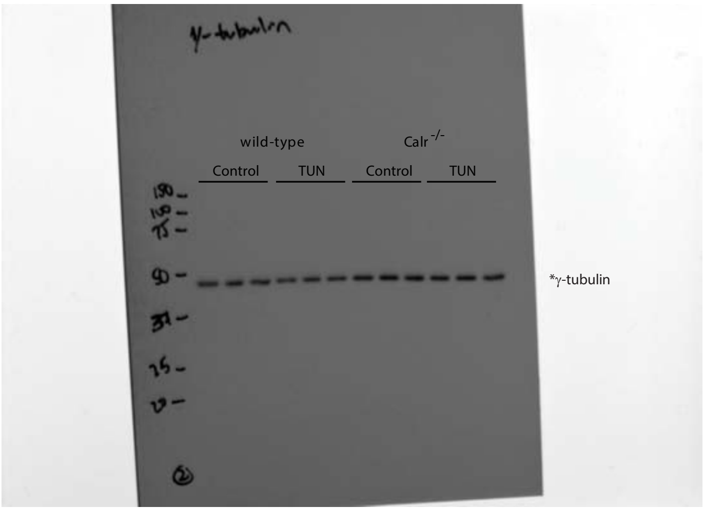

Figure S14. The full-length blots for Figure 4A

Anti-Calnexin

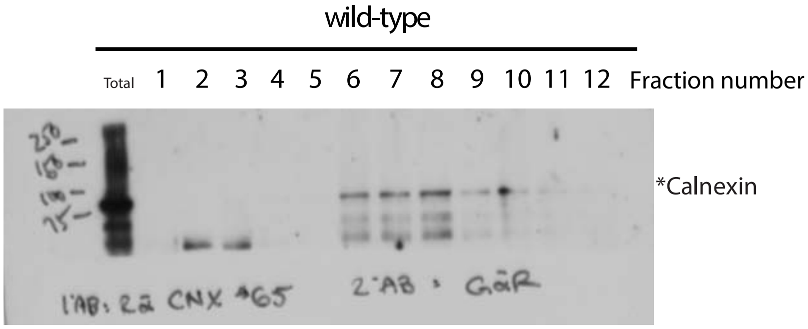

Anti-GAPDH

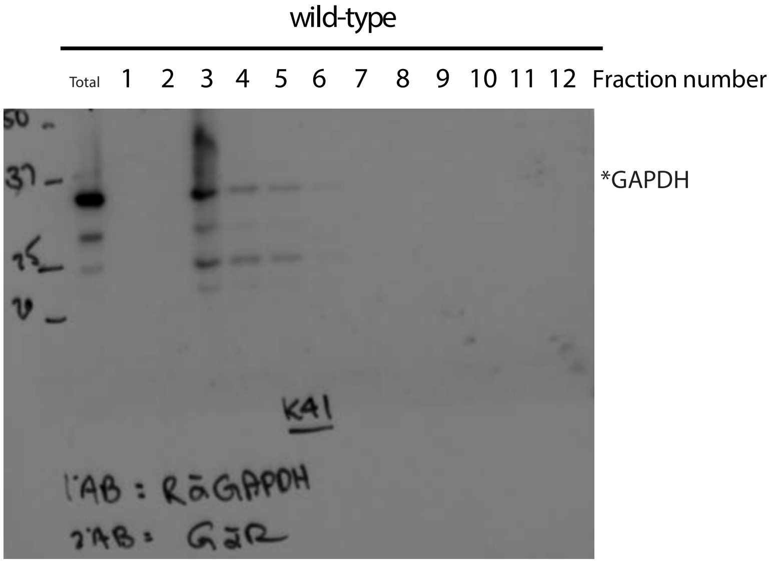

Figure S15. The full-length blots for Figure 7A

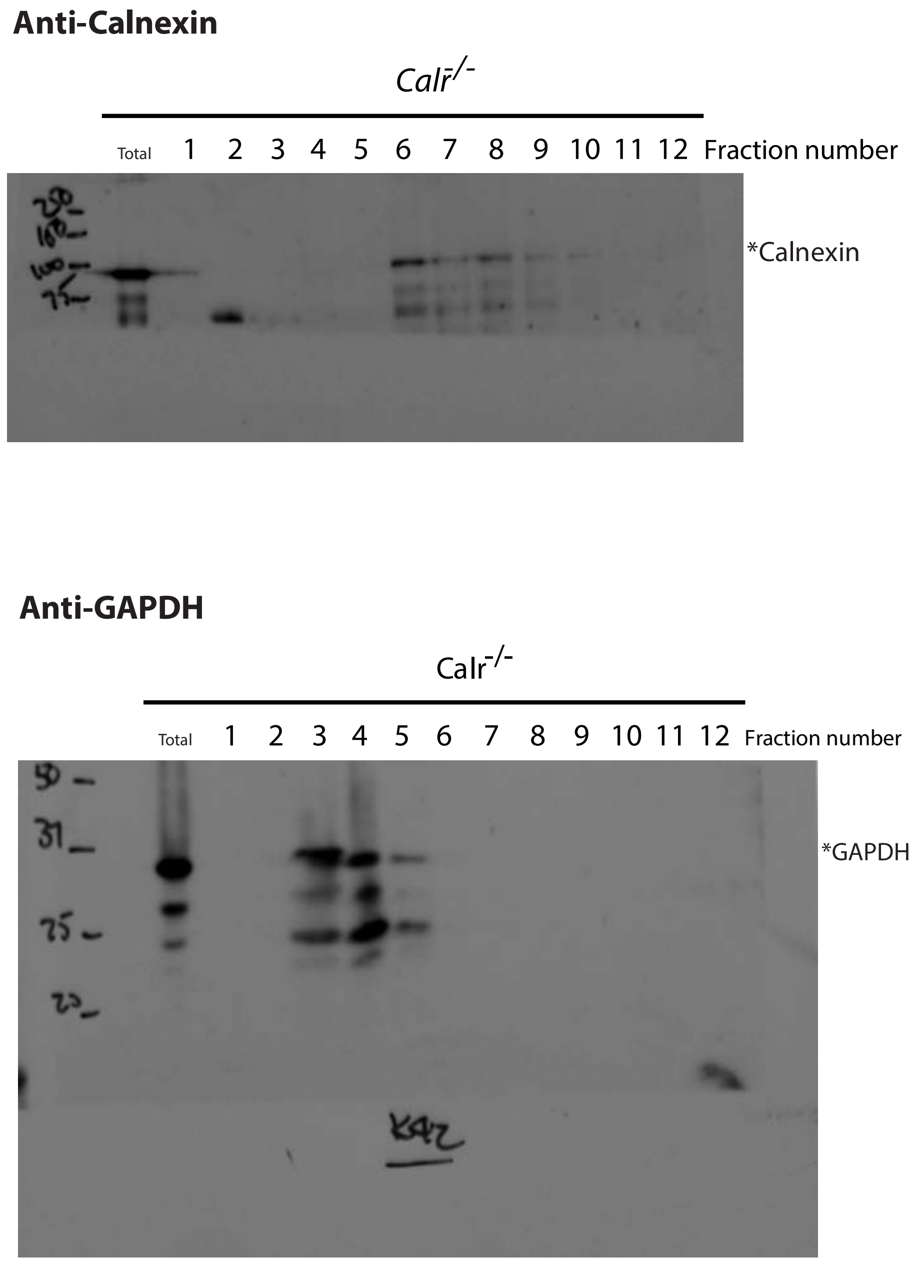

Figure S16. The full-length blots for Figure 7A

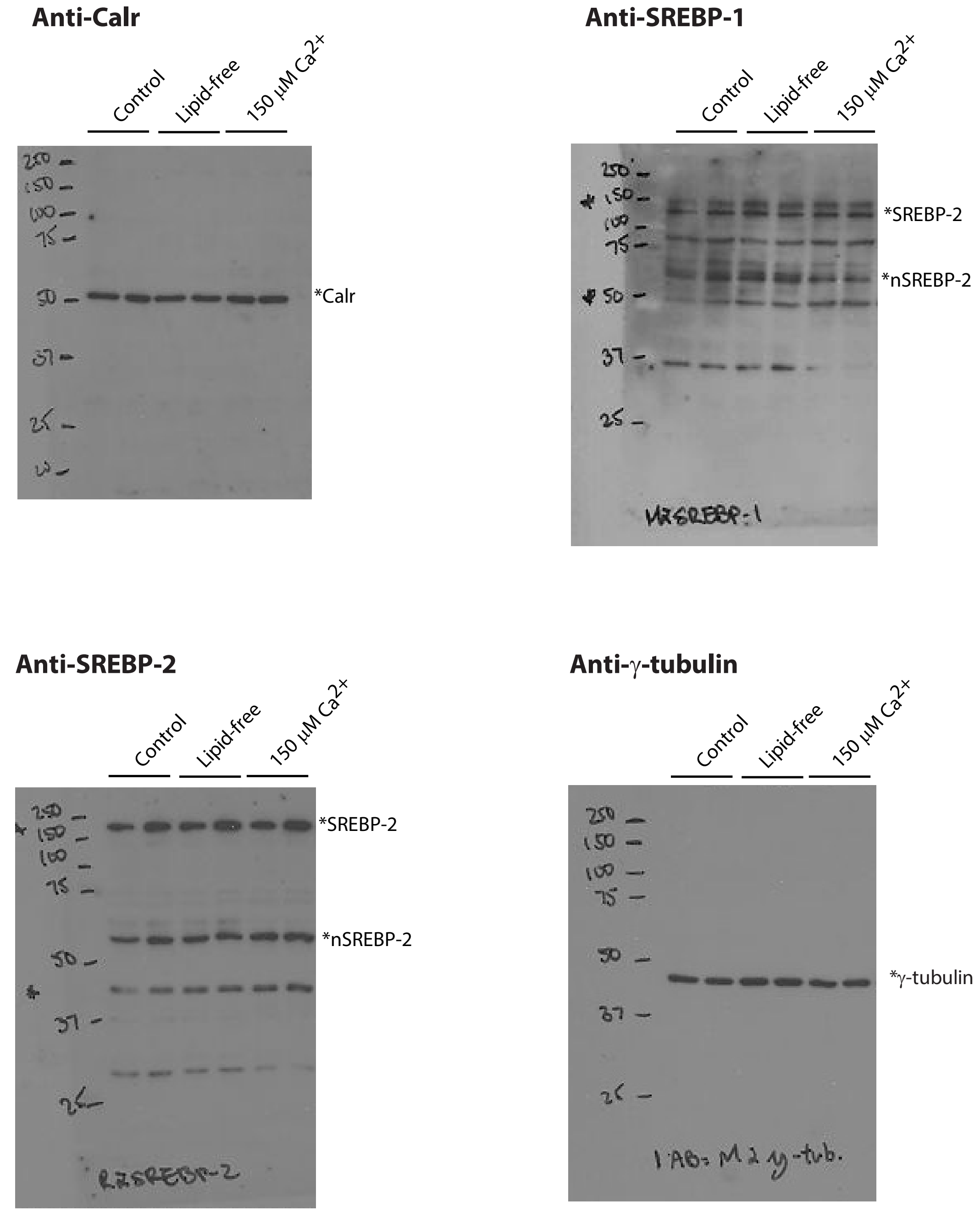

Figure S17. The full-length blots for Figure S5B
